# Lrrk promotes tau neurotoxicity through dysregulation of actin and mitochondrial dynamics

**DOI:** 10.1371/journal.pbio.2006265

**Published:** 2018-12-20

**Authors:** Farah H. Bardai, Dalila G. Ordonez, Rachel M. Bailey, Matthew Hamm, Jada Lewis, Mel B. Feany

**Affiliations:** 1 Department of Pathology, Brigham and Women’s Hospital, Harvard Medical School, Boston, Massachusetts, United States of America; 2 Department of Neuroscience and Center for Translational Research in Neurodegenerative Disease, University of Florida, Gainesville, Florida, United States of America; Columbia University, United States of America

## Abstract

Mutations in leucine-rich repeat kinase 2 (LRRK2) are the most common cause of familial Parkinson disease. Genetics and neuropathology link Parkinson disease with the microtubule-binding protein tau, but the mechanism of action of LRRK2 mutations and the molecular connection between tau and Parkinson disease are unclear. Here, we investigate the interaction of LRRK and tau in *Drosophila* and mouse models of tauopathy. We find that either increasing or decreasing the level of fly Lrrk enhances tau neurotoxicity, which is further exacerbated by expressing Lrrk with dominantly acting Parkinson disease—associated mutations. At the cellular level, altering Lrrk expression promotes tau neurotoxicity via excess stabilization of filamentous actin (F-actin) and subsequent mislocalization of the critical mitochondrial fission protein dynamin-1-like protein (Drp1). Biochemically, monomeric LRRK2 exhibits actin-severing activity, which is reduced as increasing concentrations of wild-type LRRK2, or expression of mutant forms of LRRK2 promote oligomerization of the protein. Overall, our findings provide a potential mechanistic basis for a dominant negative mechanism in LRRK2-mediated Parkinson disease, suggest a common molecular pathway with other familial forms of Parkinson disease linked to abnormalities of mitochondrial dynamics and quality control, and raise the possibility of new therapeutic approaches to Parkinson disease and related disorders.

## Author summary

Parkinson disease is a common and debilitating neurodegenerative disorder. Family and larger population-based studies have revealed a number of genes important in the development and progression of the disease, but specific mechanisms linking these seemingly unrelated genes and proteins with specific cellular pathways within affected neurons have often remained elusive. Here, we connect the pathobiology of two proteins associated with Parkinson disease through genetics and neuropathology in patients, leucine-rich repeat kinase 2 (LRRK2) and tau. Using a combination of biochemistry, cell biology, and genetic manipulation in fruit fly and mouse models, we show that LRRK2 and tau interact through the actin cytoskeleton and thereby control mitochondrial dynamics. Specifically, we find that LRRK2 can directly sever actin filaments in purified protein preparations in vitro. Consistent with our biochemical results, we see that altering the levels of LRRK changes the amount of filamentous or polymerized actin within neurons in the brain. We explore the therapeutic implications of our findings by demonstrating that decreasing polymerized actin with drugs can rescue neuronal death in animal models of disease. Our findings also link the biology of LRRK2 and tau with less common forms of familial Parkinson disease caused by mutations in the genes encoding parkin and phosphatase and tensin homolog induced putative kinase 1 (PINK1), proteins, which have previously been implicated in mitochondrial dynamics and function.

## Introduction

Parkinson disease is the second most common neurodegenerative disorder, following Alzheimer disease. Parkinson disease has a prevalence of approximately 1% at age 65, which rises to nearly 5% by age 85 [1,2]. Particularly given the increasing age of the United States population, Parkinson disease represents a significant economic burden to the healthcare system and to patients and their caregivers. There are currently no treatments that alter the course of this progressive and debilitating disorder. Parkinson disease has classically been defined as a movement disorder with loss of dopaminergic neurons from the substantia nigra and the pathological finding of aggregated α-synuclein in Lewy bodies within affected neurons. More generally, Parkinson disease belongs to a larger group of neurodegenerative parkinsonian syndromes. These parkinsonian syndromes include progressive supranuclear palsy and corticobasal degeneration, degenerative disorders characterized pathologically by abnormal aggregation and deposition of the microtubule-binding protein tau into neurofibrillary inclusions in neurons, and glia [3,4]. Diseases manifesting pathologically by neurofibrillary tau pathology are termed “tauopathies.” While parkinsonian disorders are typically regarded in terms of motor dysfunction, more recently, there has been increased recognition of nonmotor features, including psychiatric, cognitive, and autonomic dysfunction, which reflect system degenerations outside of nigrostriatial pathways and contribute significantly to patient morbidity [4–8].

Most Parkinson disease is apparently sporadic, but penetrant single-gene mutations can give rise to the disorder, and analysis of the function of encoded proteins has significantly advanced our understanding of the molecular pathogenesis of the disease [9,10]. Mutations in leucine-rich repeat kinase 2 (LRRK2), a large multidomain protein kinase of as yet incompletely understood function, represent the most common genetic cause of Parkinson disease and predispose to sporadic Parkinson disease as well [11,12]. Clinically, patients can present as Parkinson disease, Parkinson disease dementia, or dementia with Lewy bodies. Pathologically, LRRK2-associated disorders can be accompanied by a variety of neuropathologies, including aggregation and deposition of the microtubule-binding protein tau [13,14]. Mutations in the locus encoding tau have been linked to Parkinson disease [15–17], further connecting the biology of tau and sporadic Parkinson disease. We have previously demonstrated that binding and abnormal stabilization of actin by tau is an important mediator of neurotoxicity in vivo [18]. In tauopathy models, excess stabilization of actin leads to altered mitochondrial dynamics through mislocalization of the critical mitochondrial fission protein dynamin-1-like protein (Drp1). Subsequent increased accumulation of oxidative free radicals promotes premature neuronal cell death. While the function of LRRK2 in normal physiology and disease states remains incompletely characterized, proteomic, biochemical [19,20], and cell biological [21–25] studies have suggested a role in regulating actin dynamics, perhaps through direct binding to actin [19].

Motivated by the genetic, neuropathological, and cell biological connections between Parkinson disease and tau, we performed studies in our well-characterized *Drosophila* model of tauopathy, which demonstrate enhancement of tau neurotoxicity in vivo by either knockdown or overexpression of Lrrk, the single fly homolog of mammalian LRRK proteins. Tau neurotoxicity is further enhanced by expression of Lrrk carrying mutations homologous to Parkinson disease mutations in human LRRK2. These findings raise the intriguing possibility that LRRK2 mutations may act at least partially through a loss of function mechanism, perhaps via dominant negative effects in the context of autosomal dominant human mutations. In support of a possible dominant negative effect, we demonstrate inhibition of LRRK2-mediated actin severing by mutant LRRK2 in biochemical mixing experiments. In vivo, manipulation of Lrrk levels enhances tau neurotoxicity by stabilizing the actin cytoskeleton and promoting mislocalization of Drp1, leading to mitochondrial dysfunction.

## Results

### Manipulation of Lrrk enhances tau neurotoxicity

**Fig 1:**
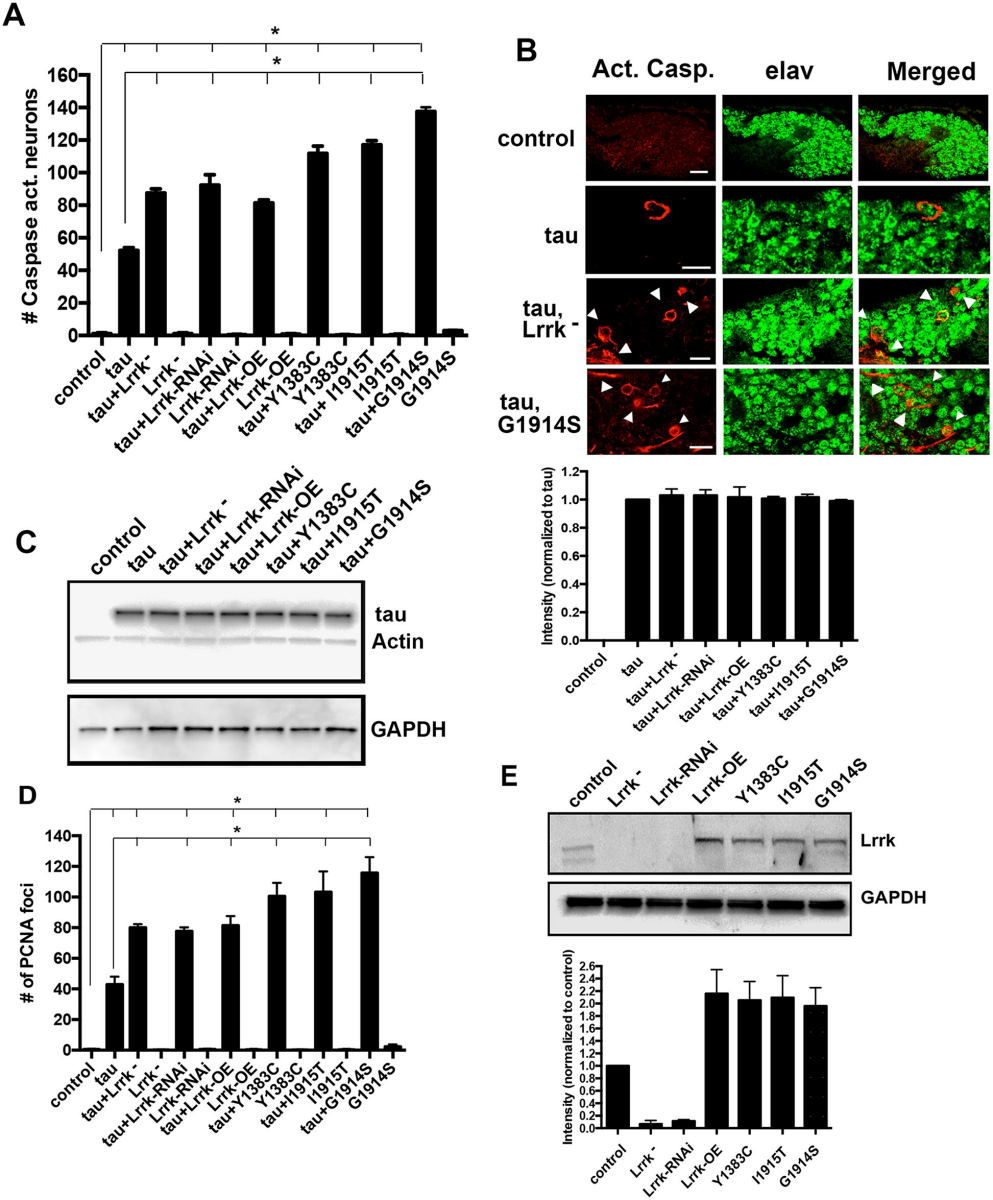
Manipulation of Lrrk enhances tau neurotoxicity. (A) Increasing or decreasing *Drosophila* Lrrk enhances the toxicity of human tau^R406W^, which is further enhanced by expressing mutant forms of Lrrk engineered to mimic Parkinson’s disease mutations. (B) Representative immunofluorescence images showing activated caspase in neurons (arrowheads, identified as neurons using anti-elav) of *Drosophila* brains. Scale bars represent 5 μm. (C) Western blot showing that manipulation of Lrrk does not change levels of transgenic tau. Quantification from three different blots is shown on the right panel. ns: not significant. Blots are reprobed for actin and GAPDH to illustrate equivalent protein loading. (D) Cell cycle activation in postmitotic neurons as monitored by PCNA staining mirrors the pattern observed for caspase activation. (E) Western blot showing levels of *Drosophila* Lrrk protein in the brains of Lrrk mutant and overexpressing flies. Quantification from three different blots is shown on the lower panel. Blots are reprobed for actin and GAPDH to illustrate equivalent protein loading. Y1383C, I1915T, and G1914S are Lrrk mutants homologous to Parkinson disease related human LRRK2 mutants Y1699C, I2020T, and G2019S, respectively. *n* = 6 per genotype and time point for histological assessments (A, D). *P < 0.01, ANOVA with supplementary Neuman—Keuls. Control is *elav-GAL4/+; UAS-CD8-PARP-Venus/+* in A and B and *elav-GAL4/+* in the remaining panels. Flies are 10 days old. See S1 Data for individual numerical values underlying the summary data displayed in A, C, D, and E. GAPDH, glyceraldehyde-3-phosphate dehydrogenase; Lrrk, leucine-rich repeat kinase; Lrrk^-^, *Lrrke*^*03680*^; Lrrk-OE, wild-type Lrrk overexpression; PCNA, proliferating cell nuclear antigen.

Unlike vertebrates in which the presence of two highly related family members, LRRK1 and LRRK2, may complicate genetic analysis, *Drosophila* contains a single LRRK protein, Lrrk [26]. To explore a possible interaction between tau and Lrrk, we first determined if either loss of endogenous fly Lrrk or overexpression of Lrrk could influence neurotoxicity in our genetically accessible transgenic *Drosophila* model of tauopathy. Our model is based on expression of either human wild-type or the familial neurodegenerative tauopathy, frontotemporal dementia with parkinsonism linked to Chromosome 17 (FTDP-17)–associated mutant tau using the GAL4/UAS bipartite expression system with the panneuronal *elav-GAL4* driver [27]. In the current *Drosophila* studies, we expressed human tau carrying the R406W mutation, which we refer to here as “tau” for simplicity. Neuronal expression of R406W mutant tau results in a level of neurotoxicity that is easily manipulated experimentally, with good conservation of the underlying mechanisms of toxicity with the wild-type human tau [28–31]. When we reduced Lrrk levels using either a homozygous protein-null mutant *Lrrk* allele or transgenic RNAi [32], we found significant enhancement of tau neurotoxicity, as assessed by the cleavage of a transgenic caspase reporter (Fig 1A) [30,33]. Interestingly, expression of wild-type fly Lrrk also enhanced tau neurotoxicity, with a further enhancement in neurotoxicity when Lrrk harboring mutations homologous to Parkinson disease—associated mutations in human LRRK2 were expressed [32]. No significant toxicity was observed when Lrrk levels were manipulated in the absence of transgenic human tau (Fig 1A). Caspase activation within neurons was confirmed by costaining with the neuronal marker elav (Fig 1B). Gain or loss of Lrrk function did not modulate toxicity by simply altering levels of transgenic tau, as determined by western blot analysis (Fig 1C).

We have previously shown that cell death occurs through inappropriate activation of the cell cycle in our tauopathy model. Cell cycle activation can be monitored by expression of proliferating cell nuclear antigen (PCNA) [28]. Aberrant cell cycle activation in tau transgenic flies with Lrrk manipulation paralleled neurotoxicity, as monitored by caspase activation, supporting a role for cell cycle activation downstream of Lrrk-mediated tau neurotoxicity (Fig 1D). To ensure that the enhancement in toxicity with the Lrrk mutants was not due to greater levels of overexpression, we monitored the expression levels of wild-type and mutant Lrrk and found that wild-type and mutant Lrrk proteins were expressed at similar levels (Fig 1E).

To examine the specificity of the interaction between Lrrk and tau, we examined an unrelated model of age-dependent neurodegeneration in *Drosophila*. We expressed mutant SCA3, a polyglutamine-expanded protein linked to spinocerebellar ataxia type 3 (Machado—Joseph disease), using the panneuronal driver *elav-GAL4* and assessed Kenyon neuron degeneration, as we have described previously [29,34]. There was no alteration of mutant SCA3 neurotoxicity with manipulation of Lrrk levels, supporting a specific effect on the pathways mediating tau neurotoxicity (S1 Fig).

To investigate the role of Lrrk kinase activity in enhancement of tau neurotoxicity, we expressed a mutant form of *Drosophila* Lrrk carrying three point mutations (K1781M, D1882A, and D1912A, analogous to K1906M, D1994A, and D2017A in human LRRK2; Lrrk-3KD), which are predicted to disrupt ATP binding [32,35]. Expression of Lrrk-3KD was less effective at enhancing tau neurotoxicity compared to wild-type Lrrk (S1 Fig), consistent with a contribution of kinase activity to the toxic effects of Lrrk in tau transgenic flies.

### Manipulation of Lrrk enhances the over stabilization of the actin cytoskeleton caused by tau

**Fig 2:**
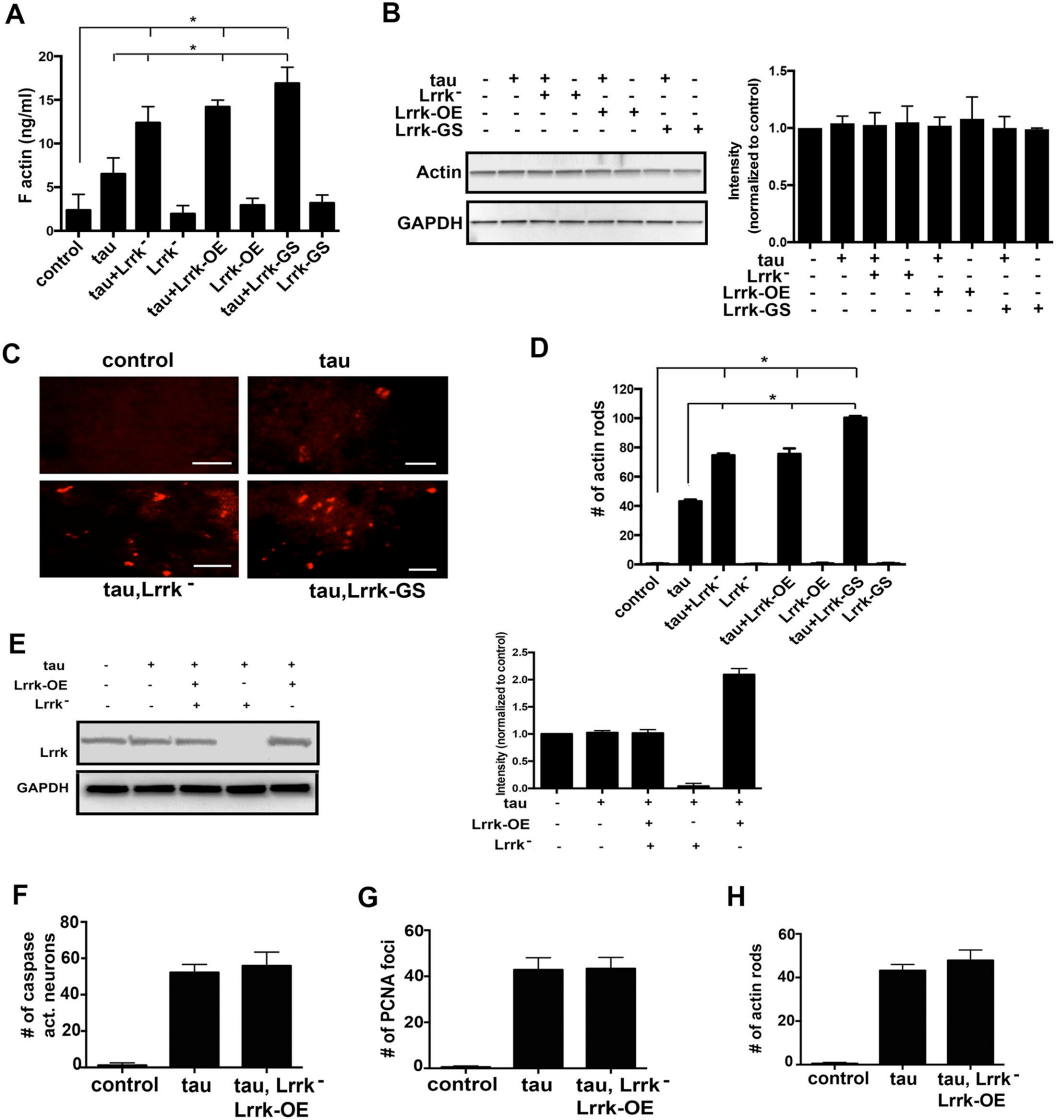
Manipulation of Lrrk enhances the excess stabilization of the actin cytoskeleton caused by tau. (A) ELISA specific for F-actin shows an increase in F-actin levels in tau transgenic flies with further increase when Lrrk levels are increased or decreased or when Lrrk-GS is expressed. *n* = 3. (B) Western blot demonstrating equivalent levels of total actin and total protein, as illustrated by GAPDH, in the genotypes studied. Quantification of actin intensity from three blots is shown on the right panel. (C) Immunofluorescence microscopy images of sections of the indicated genotypes flies stained for actin illustrating actin rods. Images shown are of cortical Kenyon neurons in the mushroom bodies. Scale bars represent 10 μm. (D) The number of actin-rich rod-like structures (actin rods) present in sections from tau transgenic flies is increased when Lrrk levels are increased or decreased. (E) Western blot showing that Lrrk overexpression in a *Lrrk* mutant background results in Lrrk levels similar to those in wild-type control animals. The blot is reprobed for GAPDH to illustrate equivalent protein levels. Quantification from three different blots is shown on the right panel. (F) Expression of wild-type Lrrk in tau transgenic flies in the *Lrrk* mutant background does not increase the number of neurons with activated caspase, indicating no increase of neuronal toxicity. (G) There is no increase in cell cycle activation in postmitotic neurons when Lrrk is expressed with tau in flies in the *Lrrk* mutant background. (H) There is no incr ease in the number of actin rods when Lrrk is expressed with tau in flies with *Lrrk* mutant background. *n* = 6 per genotype and time point for histological analyses. *P < 0.01, ANOVA with supplementary Neuman—Keuls. Control is *elav-GAL4/+*; *UAS-CD8-PARP-Venus/+* in F, and *elav-GAL4/+* in the remainder of the panels. Flies are 10 days old. See S1 Data for individual numerical values underlying the summary data displayed in A, B, and D—H. ELISA, enzyme-linked immunosorbent assays; F-actin, filamentous actin; GAPDH, glyceraldehyde-3-phosphate dehydrogenase; Lrrk, leucine-rich repeat kinase; Lrrk-GS, Lrrk carrying the G1914S mutation.

We next addressed the cellular mechanism by which Lrrk enhances tau neurotoxicity. We have previously shown that tau binds to and stabilizes actin and that actin stabilization is critical for tau neurotoxicity using genetic analyses [36]. LRKK2 has also been implicated in regulation of the actin cytoskeleton [19–24]. We thus assessed the effect of Lrrk on actin dynamics. To monitor stabilization of the actin cytoskeleton in multiple genotypes in parallel, we performed filamentous actin (F-actin) enzyme-linked immunosorbent assays (ELISA) on brain lysates. We examined brains of tau transgenic flies homozygous for the Lrrk mutant allele or expressing either wild-type Lrrk or Lrrk carrying the G1914S mutation (Lrrk-GS), the mutation homologous to the most common Parkinson disease—associated mutation in human LRRK2 (G2019S). There was a robust increase in the levels of F-actin in the brains of tau transgenic flies with Lrrk manipulation (Fig 2A and 2B). We have previously demonstrated the presence of actin-rich rods similar to Hirano bodies in the brains of tau transgenic flies [36]. We found a significant increase in the number of actin rods in the brains of tau transgenic flies with Lrrk manipulation (Fig 2C and 2D).

To ensure that the effects of overexpressing Lrrk reflected the function of the endogenous protein, we expressed wild-type Lrrk along with tau in a *Lrrk* mutant background. Total levels of Lrrk were normalized by removing endogenous Lrrk (Fig 2E). Accordingly, the enhancement of caspase activation (Fig 2F), cell cycle activation (Fig 2G), and the number of actin rods (Fig 2H) were all rescued by reducing Lrrk levels. Note that manipulating Lrrk expression did not alter expression of transgenic tau (Fig 1C). The enhancement in neurotoxicity and the number of actin rods seen in the *Lrrk* mutant animals were also rescued by mutant Lrrk-GS, indicating that the disease-linked mutant retains the activity of wild-type Lrrk (S2 Fig).

We also assessed the ability of human LRRK2 to enhance tau neurotoxicity in our *Drosophila* model. Expressing human LRRK2 with tau enhanced the neurotoxicity of tau, as assessed by caspase activation (S2 Fig). Cell cycle activation was also increased with LRRK2 coexpression, as were the number of actin rods (S2 Fig), suggesting that the same pathways of toxicity are activated with expression of human LRRK2 as are activated with expression of *Drosophila* Lrrk.

### Mislocalization of Drp1, mitochondrial elongation, and markers of mitochondrial dysfunction are enhanced with Lrrk manipulation

**Fig 3:**
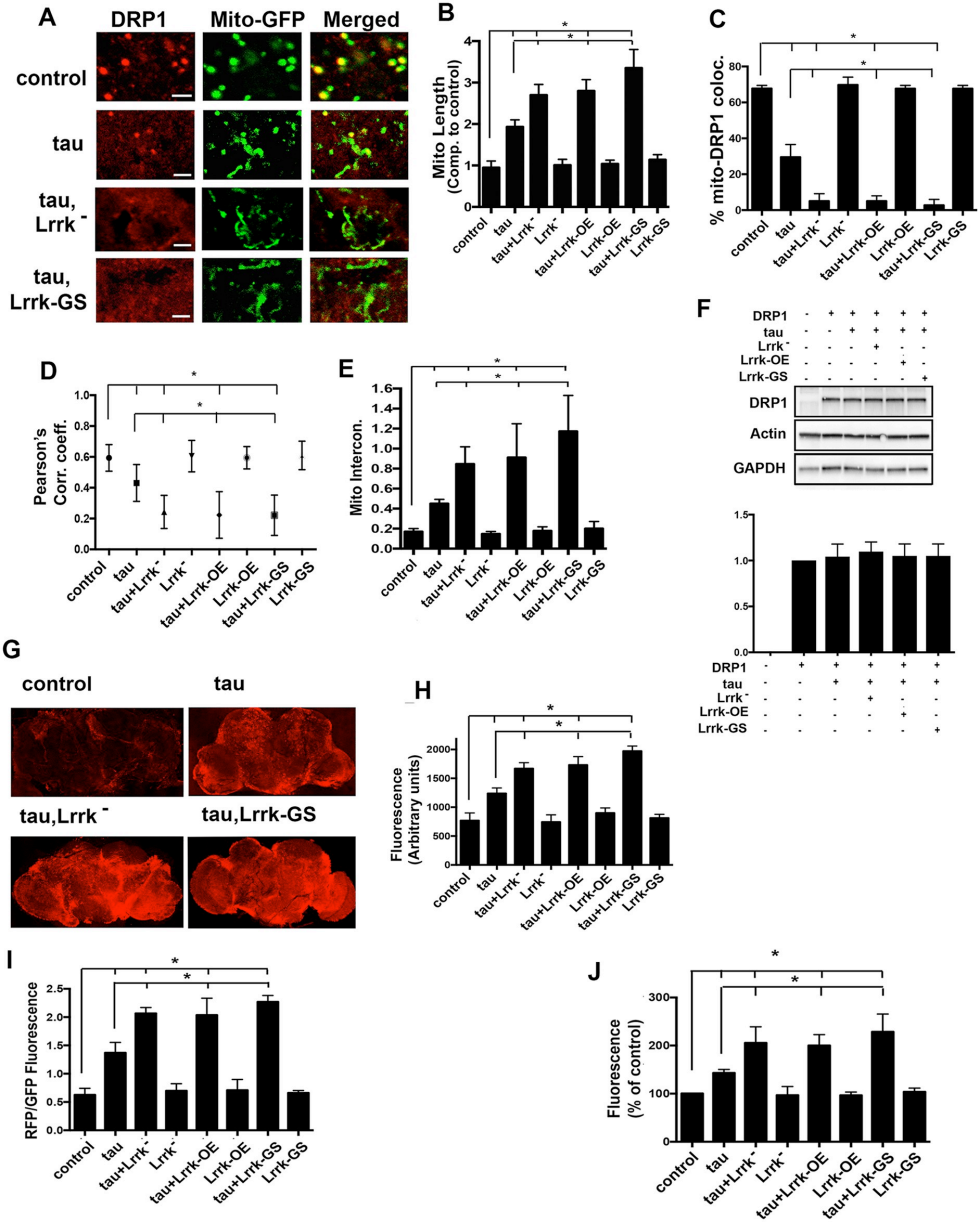
Mislocalization of Drp1, mitochondrial elongation, and mitochondrial dysfunction are enhanced by Lrrk manipulation. (A) Representative confocal images of cortical Kenyon neurons of the *Drosophila* brain sections show mislocalization of the mitochondrial fission protein Drp1 and elongated mitochondria in tau transgenic flies. Mitochondrial elongation and Drp1 mislocalization are enhanced in flies lacking or overexpressing Lrrk. Scale bars represent 2 μm. (B) Quantification of mitochondrial length shows elongated mitochondria in tau transgenic flies with modulated levels of Lrrk. (C) Quantification of the number of mitochondria colocalized with Drp1 shows reduced mitochondrial localization of Drp1 in tau transgenic flies, with further reduction with manipulation of Lrrk. (D) Measurement of Pearson’s correlation coefficient reflects reduced colocalization with genetic manipulation of tau and Lrrk. (E) Mitochondrial interconnectivity is increased with expression of tau and further increased by manipulating Lrrk expression. (F) Western blot showing equal levels of Drp1 protein in experimental genotypes as indicated. The blot is reprobed for actin and GAPDH to illustrate equivalent protein loading. The lower panel shows quantification of the Drp1 band intensity from three different blots. (G) Confocal images of whole fly brains freshly dissected and stained with the mitochondrial superoxide dye MitoSOX. Two-dimensional projection of z-stacks showing the brightest section for each sample. (H) Quantification of the fluorescence intensity for the entire brain shows an increase in superoxide levels in the mitochondria of tau transgenic flies, which is further enhanced in tau transgenic flies with altered Lrrk expression. (I) Measurement of the ratio of red to green fluorescence in flies with the *UAS-MitoTimer* reporter transgene reflects increased reporter oxidation in tau transgenic flies, which is further increased with manipulation of Lrrk expression. (J) Measurement of red fluorescence indicating the levels of ROS in the brains of flies of the indicated genotypes shows increased ROS levels in flies expressing tau, with further increases with manipulation of Lrrk expression. *n* = 6 per genotype and time point in B, C, D, E, H, I, and J. *P < 0.01, ANOVA with supplementary Neuman—Keuls. Control is *elav-GAL4/+*; *UAS-mito-GFP/+; HA-Drp1/+* in A—F, *elav-GAL4/+* in G, H, and I, and *elav-GAL4/+*; *UAS-MitoTimer/+* in J. Flies are 10 days old. See S1 Data for individual numerical values underlying the summary data displayed in B—F and H—J. Drp1, dynamin-1-like protein; GAPDH, glyceraldehyde-3-phosphate dehydrogenase; Lrrk, leucine-rich repeat kinase; mito-GFP, mitochondrially directed GFP; ns, not significant; ROS, reactive oxygen species; HA, hemagglutinin.

We have shown using genetic and biochemical methods that abnormal stabilization of actin by tau promotes mislocalization of the critical mitochondrial fission protein Drp1, leading to elongation of mitochondria [18]. To determine if there is Drp1 mislocalization and altered mitochondrial morphology in tau transgenic flies in Lrrk-modified backgrounds, we expressed two additional transgenes. To visualize mitochondria, we expressed mitochondrially directed green fluorescent protein (mito-GFP). To visualize Drp1, we used a 9.35-kb genomic rescue strain that has an in-frame FLAG-FIAsH-hemagglutinin (HA) tag after the start codon of Drp1, thus expressing tagged Drp1 under its endogenous promoter [18,37]. Manipulation of Lrrk promoted additional mitochondrial elongation in the brains of tau transgenic flies (Fig 3A and 3B). There was also a significant loss of localization of Drp1 to mitochondria in tau transgenic flies with manipulated Lrrk backgrounds (Fig 3A and 3C). The colocalization analysis was confirmed by computing the Pearson’s correlation coefficient. The average value of the Pearson’s coefficient for the cells analyzed decreased in tau transgenic flies compared to control, with an even further decrease in tau transgenic flies with Lrrk manipulations (Fig 3D), indicating an enhancement in the loss of Drp1 localization to the mitochondria. Utilizing a computational method to assess mitochondrial interconnectedness [38], we observed an increase in the degree of mitochondrial interconnectedness in tau transgenic flies with Lrrk manipulation (Fig 3E). Drp1 protein levels remained unchanged in the heads of flies with Lrrk genetic manipulation (Fig 3F), demonstrating that the loss of Drp1 localization to the mitochondria was not due to a reduction in Drp1 levels.

Since loss of dopaminergic neurons is clinically important in Parkinson disease, we examined these monoaminergic neurons in our model. Examination of tyrosine hydroxylase (TH) immunoreactive neurons in the anterior medulla of flies expressing human tau [39] revealed loss of TH-positive neurons. Loss of TH immunoreactive neurons was enhanced when Lrrk levels were reduced or if either wild-type or mutant Lrrk was expressed in tau transgenic flies (S3 Fig). Dopaminergic pathology has previously been reported with expression of human LRRK2 in transgenic *Drosophila* [40,41], although at later time points than were examined in the current study.

Drp1 mislocalization and mitochondrial elongation are accompanied by increased levels of reactive oxygen species (ROS) production in the brains of tau transgenic flies [18]. To determine if changes in mitochondrial dynamics by the manipulation of Lrrk correlate with an increase in markers of mitochondrial stress, we first used MitoSOX on freshly dissected whole brains. The MitoSOX dye permeates live cells, in which it is targeted to the mitochondria and becomes oxidized by superoxide, resulting in strong red fluorescence, which can be detected by microscopy [42]. Fluorescence in the brains of flies with manipulated Lrrk along with tau expression was greater than that in control flies (Fig 3G and 3H), consistent with elevated levels of mitochondrial superoxide. Next, we used a genetic reporter, MitoTimer, which is targeted to the mitochondria, and the fluorescence shifts from green to red on oxidation. The ratio of red to green fluorescence gives a measure of mitochondrial stress [43]. We observed an increase in oxidized MitoTimer protein in the brains of tau transgenic flies with altered Lrrk function (Fig 3I). To measure intracellular ROS, we used ROSstar 650, a hydrocyanine-based probe designed to detect specifically superoxide and hydroxyl radicals [44]. There was an increase in ROS levels in the brains of flies expressing tau with Lrrk manipulation (Fig 3J).

### Pharmacological inhibition of actin polymerization rescues neurodegeneration and mitochondrial function in tau transgenic flies

**Fig 4:**
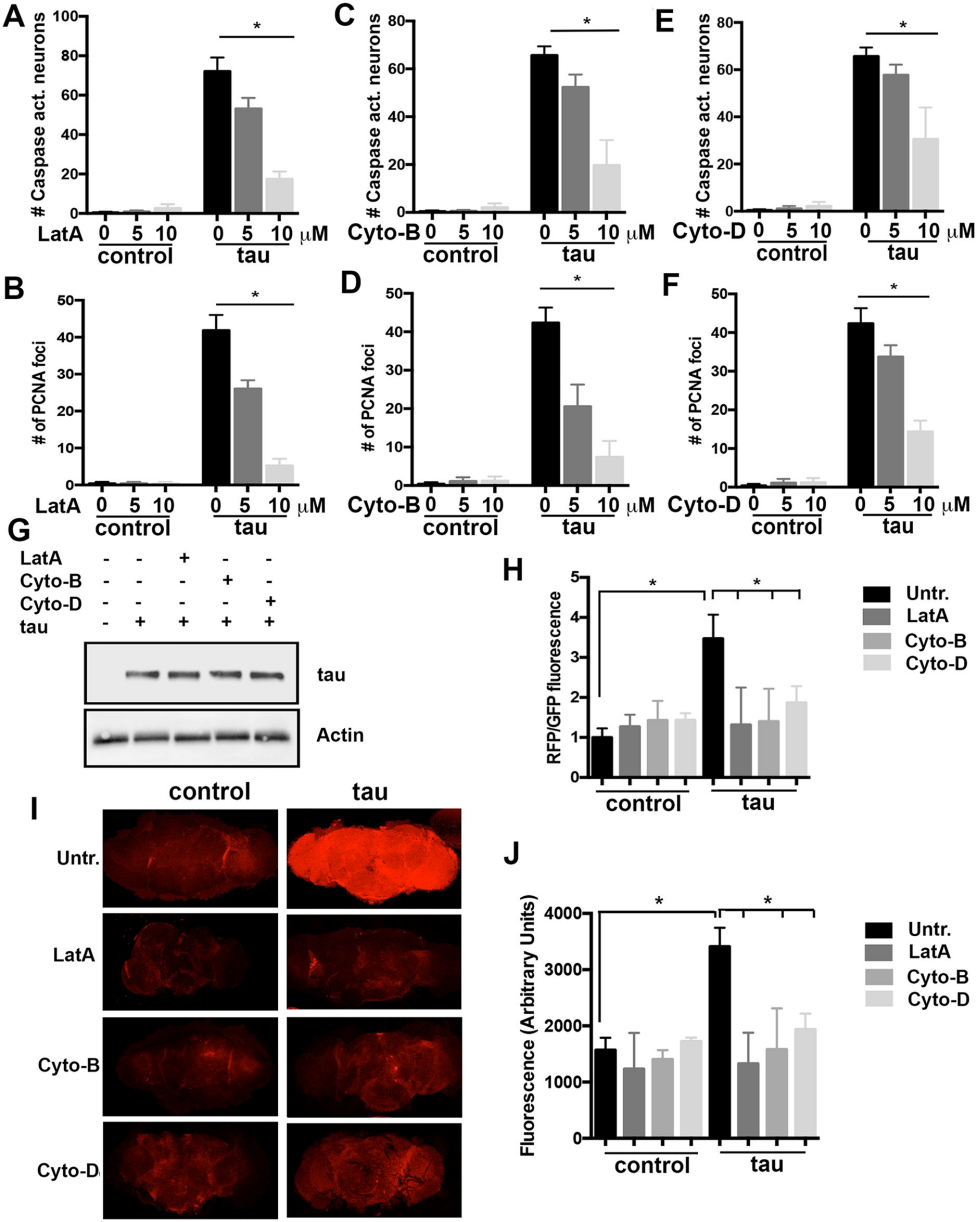
Pharmacological inhibition of actin polymerization rescues neurodegeneration and mitochondrial deficits in tau transgenic flies. (A) Dose-dependent improvement in tau neurotoxicity with feeding of the actin-depolymerizing compound LatA, as assessed by the number of activated caspase positive cells. (B) Cell cycle activation is reduced in flies treated with LatA. (C, D) Treatment with Cyto-B results in a dose-dependent reduction in caspase activation (C), and also in cell cycle activation (D). (E, F) Treatment with Cyto-D results in a significant reduction in caspase activation (E) and in cell cycle activation (F). (G) Western blot showing that drug treatments at 10 μM do not change levels of transgenic human tau. (H) Measurement of the ratio of red to green fluorescence in flies with the *UAS-MitoTimer* reporter transgene shows a reduction in reporter oxidation in tau transgenic flies treated with 10 μM of either LatA, Cyto-B, or Cyto-D. (I) Representative confocal images of either control or tau transgenic fly brains treated with actin depolymerization drugs and stained for superoxide indicator dye MitoSOX. Two-dimensional projections of z-stacks showing the brightest section for each sample. (J) Quantification of the fluorescence intensity of the entire brain shows a decrease in superoxide levels in tau transgenic flies treated with 10 μM of actin-depolymerizing drugs. *n* = 6 per genotype and treatment. *P < 0.01, ANOVA with supplementary Neuman—Keuls. Control is *elav-GAL4/+; UAS-CD8-PARP-Venus/+* (A, C, and E), *elav-GAL4/+* in B, D, F, G, I, and J, and *elav-GAL4/+*; *UAS-MitoTimer* in H. Flies are 10 days old. See S1 Data for individual numerical values underlying the summary data displayed in A—F, H, and J. Cyto-B, cytochalasin B; Cyto-D, cytochalasin D; LatA, latrunculin A.

To explore the translational potential of our findings, we treated tau transgenic flies with actin-depolymerizing drugs. Latrunculin A binds actin monomers and thereby prevents their polymerization [45]. Consistent with our prior genetic data [18,36,46], feeding flies 5 or 10 μM latrunculin A produced a robust, dose-dependent rescue of tau neurotoxicity (Fig 4A). We also observed a dose-dependent decline in cell cycle activation with oral administration of latrunculin A (Fig 4B). Cytochalasins are a widely studied family of compounds, which disrupt actin polymers [47]. We found that cytochalasin B feeding also robustly rescued neurotoxicity in tau transgenic flies; cytochalasin D was moderately effective (Fig 4C and 4E). We observed rescue of cell cycle activation by the two compounds, with cytochalasin B having greater efficacy than cytochalasin D (Fig 4D and 4F). All three compounds were also able to rescue the enhanced neurotoxicity of tau expressed in a *Lrrk* mutant background or with increased Lrrk expression (S4 Fig). Latrunculin A, cytochalasin B, or cytochalasin D did not alter the levels of tau (Fig 4G). Actin-targeting compounds also rescued the mitochondrial dysfunction in tau transgenic flies, as indicated by a reduction in the oxidation of the MitoTimer protein (Fig 4H) and decreased MitoSOX fluorescence (Fig 4I and 4J).

### Enhancement of neurodegeneration, actin rod formation, Drp1 mislocalization, and mitochondrial elongation in mice expressing human tau and human LRRK2

**Fig 5:**
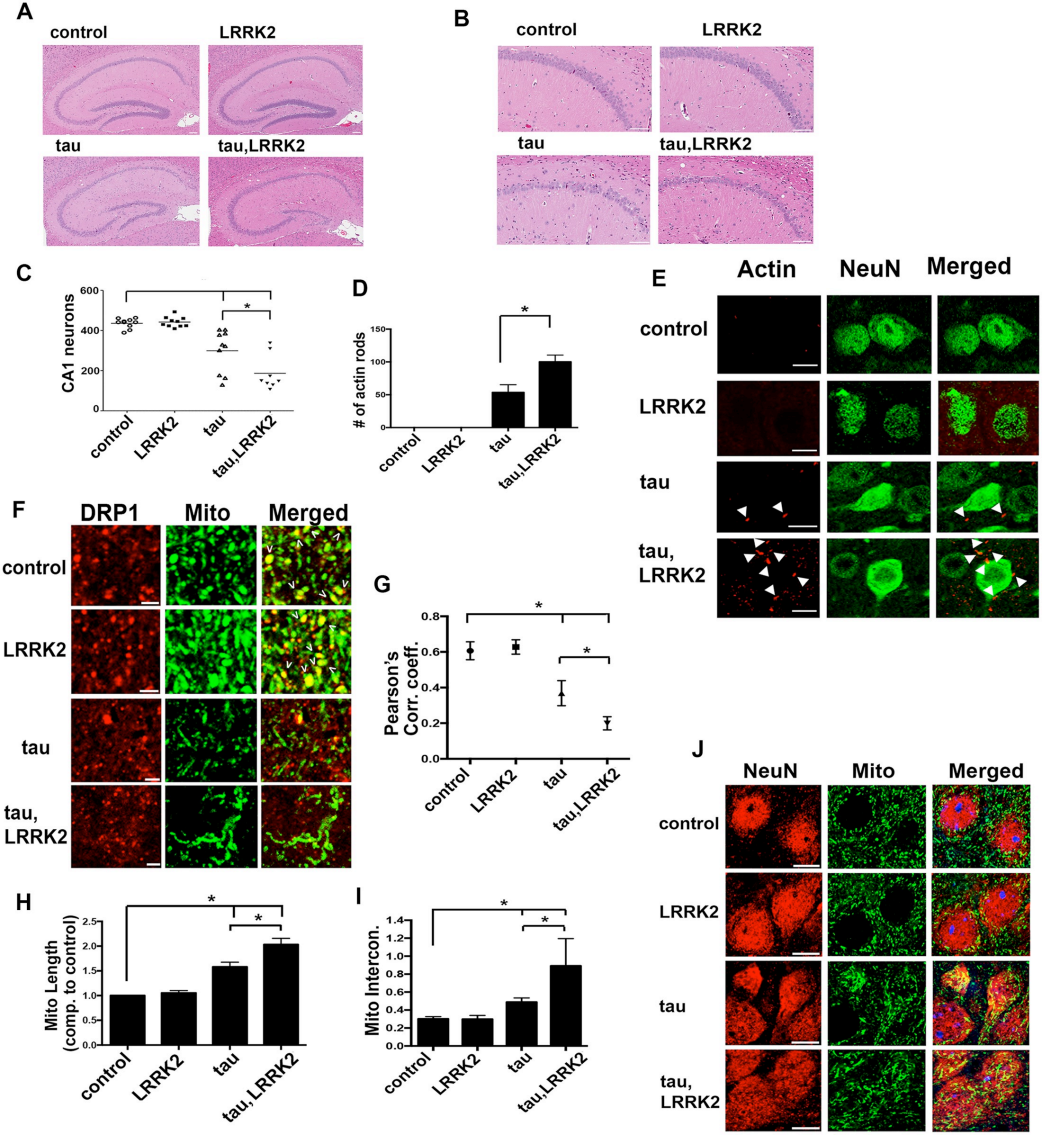
Enhancement of neurodegeneration, actin rod formation, Drp1 mislocalization, and mitochondrial elongation in mice expressing human tau and human LRRK2. (A) H&E staining of sagittal brain sections of mice shows neuronal loss in the hippocampus of transgenic mice expressing human tau (tau^P301L^), which is enhanced in mice overexpressing wild-type human LRRK2 and human tau (tau—LRRK2). (B) Higher magnification of the CA1 region. Scale bars in A, B represent 100 μm. (C) Quantification of the number of neurons in the CA1 region in the brain sections of mice with the indicated genotypes. *n* = 10 per genotype. Overall *P* value (ANOVA) for difference is <0.0001. Bonferroni multiple comparison posthoc test shows that each group is significantly different from all others with a *P* value lower than 0.01 across the board. The single exception is the comparison of nontransgenic and LRRK2 groups, which are not significantly different from one another. (D) Quantification of actin-rich rod-like structures in brain sections of mice shows an increase in tau—LRRK2 mice. (E) Representative immunofluorescence images showing actin rods in the brains of transgenic mice. Scale bars represent 10 μm. (F) Immunofluorescence images of mouse hippocampal neurons stained for ATPVa to visualize the mitochondria and Drp1 show a decrease in Drp1 localization to mitochondria in human tau transgenic mice and a further reduction in tau—LRRK2 mice. G) Pearson’s correlation coefficient calculated as a measure of colocalization. (H) Quantification of mitochondrial length in the neuronal cell bodies reveals an increase in average mitochondrial length in human tau transgenic mice, which is further increased in tau-LRRK2 mice. (I) Mitochondrial interconnectedness is increased in human tau transgenic mice, and further increased in tau—LRRK2 mice. (J) Immunofluorescent images of mouse brain sections stained with NeuN to visualize the hippocampal pyramidal neurons and ATPVa to demonstrate mitochondrial morphology. Scale bars represent 10 μm. *n* = 5 per genotype. *P < 0.01, ANOVA with supplementary Neuman—Keuls for D, G, H, and I. Controls are age-matched nontransgenic mice. Mice are 5.5 months old. See S1 Data for individual numerical values underlying the summary data displayed in C, D, G, H, and I. H&E, hematoxylin and eosin; ATPVa, vacuolar protein-ATPase A-subunit; Drp1, dynamin-1-like protein; LRRK2, leucine-rich repeat kinase 2.

To investigate the interaction of LRRK2 and tau in a mammalian system, we used transgenic mice expressing FTDP-17-associated P301L mutant human tau in the forebrain via a calcium/calmodulin-dependent protein kinase II reverse transactivator (CAMKIIα-tTA) transgene [48] crossed to wild-type LRRK2 bacterial artificial chromosome (BAC) transgenic mice. These tau transgenic mice have robust neurodegeneration, including in the cornu ammonis 1 (CA1) region of the hippocampus, reflecting expression of the tau transgene [49] in an anatomic area involved in common tauopathies and α-synucleinopathies [50–52]. The LRRK2 BAC mice have no overt phenotypic or pathological abnormalities at baseline [53]. We observed increased neuronal loss in the CA1 region of transgenic mice expressing both tau and LRRK2 (Fig 5A–5C). These human tau transgenic mice also have actin-rich rods similar to Hirano bodies in their brains [36]. The number of actin rods in the brains of mice expressing both tau and LRRK2 was increased (Fig 5D and 5E). Rods were most abundant in the deep gray nuclei, including the basal ganglia and thalamus, consistent with prior observations [36].

We next assessed Drp1 localization and mitochondrial morphology in the murine model. We have previously demonstrated mislocalization of Drp1 and elongated mitochondria in these tau transgenic mice [18]. The failure of Drp1 to localize to the mitochondria was enhanced in transgenic mice expressing human tau together with human LRRK2 (Fig 5F and 5G). The average length and the interconnectedness of the mitochondria in hippocampal pyramidal neuronal cell bodies of mice expressing both tau and LRRK2 were increased (Fig 5H and 5J, S5 Fig). We have previously shown that the expression levels of transgenic tau are not affected by the expression of LRRK2 and that LRRK2 expression from the BAC transgenic construct remains robust at the 5.5 month time point examined in the current study [48].

### Lrrk enhances actin depolymerization by severing F-actin filaments

**Fig 6:**
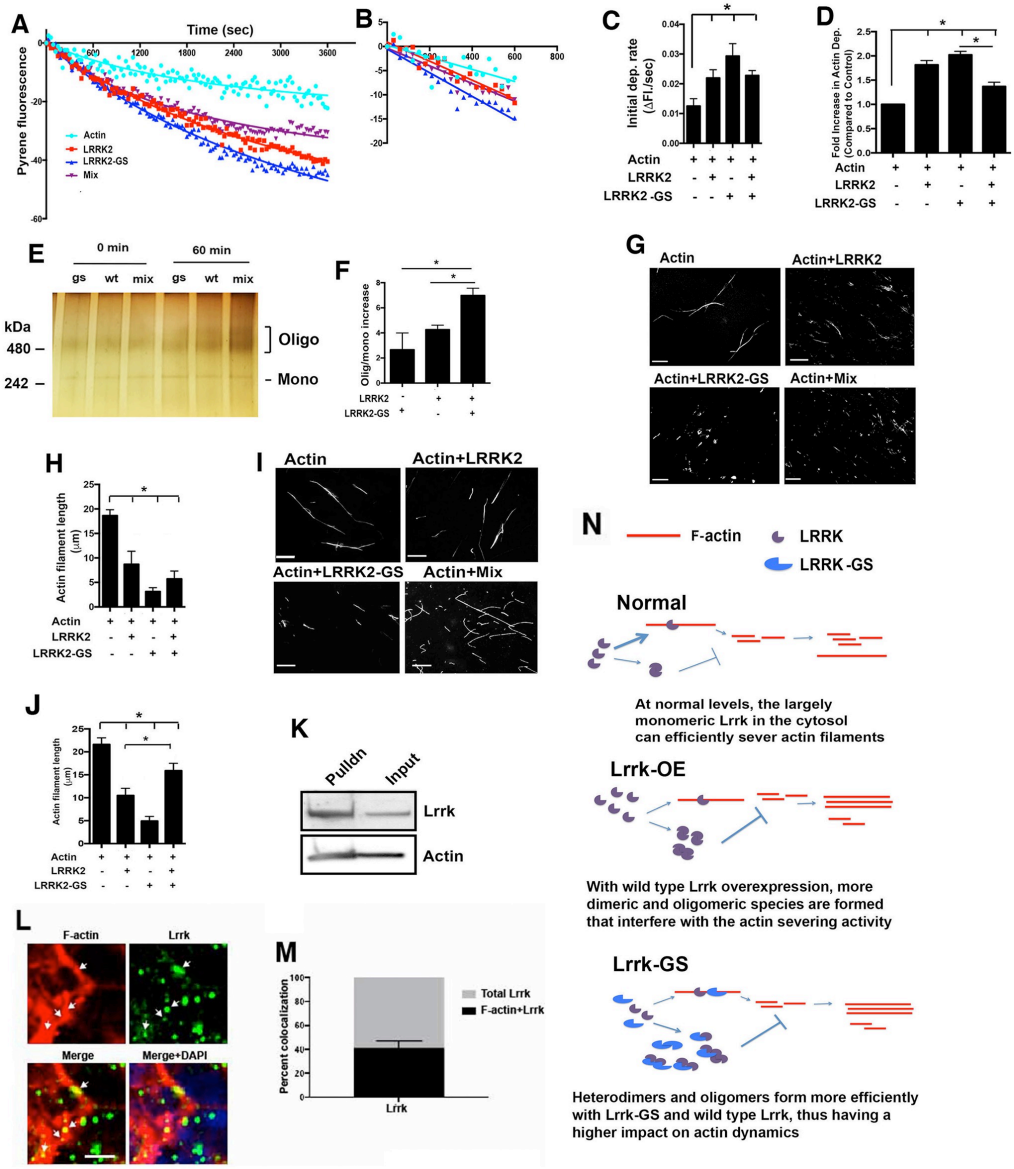
Lrrk enhances actin depolymerization by severing F-actin filaments. (A) Actin depolymerization assay using pyrene-labeled actin (4 μM) and recombinant LRRK2 proteins, either wild type, LRRK2-GS, or 1:1 mixture of the two (mix) used at 1 nM shows an enhancement of actin depolymerization by LRRK2. (B) Initial 10 minutes of the actin depolymerization assay showing the linear phase of depolymerization. (C) Initial rates of depolymerization calculated from the liner portion of the depolymerization curves. (D) Fold increase in depolymerization of actin filaments in the presence of LRRK2, LRRK2-GS, or the mix at 60 minutes. (E) Native gel with silver stain showing oligomerization states of LRRK2 proteins with 0 or 60 minutes incubation at room temperature. (F) Graph showing the percentage increase in the oligomer/monomer ratio in 60 minutes for the specified samples after quantification of three different gels. (G) Representative pictures of the results of actin severing assays on in vitro polymerized actin filaments with either wild-type LRRK2, LRRK2-GS, or the mix at 1 nM. (H) Quantification of the actin severing assay showing severing activity by LRRK2, LRRK2-GS, or the mix after a two-minute incubation with polymerized actin filaments. (I) Representative pictures of the results of the actin severing assays on in vitro polymerized F-actin performed after incubating the indicated LRRK2 proteins for 1 hour. (J) Quantification of the actin severing assays performed after preincubation of the LRRK2 proteins. (K) Biotinylated phalloidin precipitation of F-actin from control (genotype: *elav-GAL4/+*) *Drosophila* heads shows that Lrrk interacts with F-actin in vivo. (L, M) Immunofluorescence images of freshly dissected brains from flies expressing HA-tagged Lrrk from the endogenous promoter (genotype: *elav-GAL4/+*; *Lrrk*^*HA*^/+) and costained with an anti-HA antibody and fluorescent phalloidin show colocalization of Lrrk with F-actin. *n* = 3 for all experiments. *P < 0.01, ANOVA with supplementary Neuman—Keuls. Flies are 1–3 days of age. Scale bars represent 10 μm for G and I and 5 μm for L. See S1 Data for individual numerical values underlying the summary data displayed in A—D, F, H, J, and M. (N) Model of the proposed effects of Lrrk oligomerization state on actin severing activity. At normal levels, Lrrk exists primarily in the monomeric state and severs actin filaments efficiently. When Lrrk is overexpressed, higher concentrations of Lrrk lead to the formation of oligomeric species that have reduced or absent actin severing activity. In the presence of mutant Lrrk, oligomers form with increased efficiency, further reducing actin-severing activity. F-actin, filamentous actin; HA, hemagglutinin; LRRK, leucine-rich repeat kinase; LRRK2-GS, LRRK2-G2019S.

To explore the mechanism by which Lrrk influences actin dynamics, we performed in vitro depolymerization assays using pyrene-labeled actin and recombinant human wild-type LRRK2 and LRRK2 with the G2019S mutation (LRRK2-GS). Since LRRK2 mutations causing Parkinson disease can act in an autosomal dominant fashion, with both wild-type and mutant protein being present, we also included a mixture of wild-type and mutant recombinant proteins in a 1:1 ratio (mix) in our experiments. The LRRK2 proteins were used at 1 nM, a concentration that is comparable to estimated endogenous levels of LRRK2 in brain [54]. Wild-type LRRK2 and LRRK2-GS as well as the mix enhanced the depolymerization of actin filaments to varying degrees (Fig 6A). The initial depolymerization rate, measured by computing the slope of the depolymerization curve in the first 10 minutes, was similar for the wild type and the mix (Fig 6B and 6C). However, the fold increase in depolymerization at the end of 1 hour was greater for the wild type and LRRK2-GS compared to the mix, indicating a misregulation of actin dynamics in the presence of both wild type and LRRK2-GS (Fig 6D). To investigate the mechanism underlying altered behavior of the mixture biochemically, we examined the oligomerization state of the proteins. We incubated the recombinant LRRK2 proteins at room temperature for 1 hour and visualized oligomeric species on a native gel by silver staining. We observed an increase in the ratio of oligomeric to monomeric species over time, with greater oligomerization in the mix compared to the wild type or LRRK2-GS alone (Fig 6E and 6F).

To assess Lrrk oligomerization in vivo, we used a *Lrrk*^*HA*^ knockin line in which HA-tagged Lrrk is expressed from its endogenous promotor [55]. We expressed transgenic wild-type or mutant Lrrk-GS in flies also carrying one *Lrrk*^*HA*^ allele and used head lysates to perform native polyacrylamide gel electrophoresis (PAGE). Probing the resultant western blots with an antibody directed to HA revealed dimerization as well as higher-order oligomerization in flies overexpressing wild-type Lrrk protein, which was enhanced in the flies that express Lrrk-GS (S5 Fig).

To determine how LRRK2 promotes depolymerization of actin filaments, we assayed actin filament—severing activity. We observed that wild-type LRRK2, LRRK2-GS, as well as the mix could sever polymerized F-actin filaments, with the severing activity of the mix being intermediate between that of the wild type and LRRK2-GS (Fig 6G and 6H). The severing experiment in Fig 6G and 6H was performed with freshly prepared LRRK2 and actin, thus reflecting the initial phase of the depolymerization studies (Fig 6B and 6C), in which we expect more monomeric species to be present in all the samples (Fig 6E). When we repeated the actin severing assay after incubating LRRK2, LRRK2-GS, and the mix at room temperature for 1 hour, thus allowing oligomer formation, we saw a significant reduction in the actin-severing activity of the mix (Fig 6I and 6J). The reduction in the severing activity in the mixture of wild-type and mutant LRRK2 is consistent with increased oligomer formation in the mix (Fig 6E and 6F), suggesting a link between the actin-severing activity and the oligomeric state of LRRK2. To confirm that Lrrk and F-actin interact in vivo, we precipitated F-actin biochemically using biotinylated phalloidin. We observed coprecipitation of Lrrk with F-actin in head homogenates from flies (Fig 6K). Similarly, Lrrk and F-actin colocalized in sections from fly brains (Fig 6L and 6M). Consistent with a close interaction between F-actin and mitochondria [18,56–58], Lrrk also colocalized with mitochondria in fly brains (S6 Fig).

The model in Fig 6N predicts that strong loss of Lrrk function should promote F-actin stabilization, even in the absence of transgenic human tau expression. While we did not observe clear alterations in the actin cytoskeleton (Fig 2) or mitochondria (Fig 3) in Lrrk mutant animals at the 10-day time point we typically use to assess tau neurotoxicity in our model (Fig 1) [18,28,36], we did observe modest but statistically significant abnormalities in older animals. When we examined actin stabilization in flies with loss of endogenous Lrrk aged to 20 days, we observed actin stabilization as assessed by phalloidin staining and precipitation of F-actin using biotinylated phalloidin (S7 Fig). Increased numbers of actin-rich rods were also present in older Lrrk mutant animals (S7 Fig). As predicted (Fig 6N), Drp1 mislocalization and mitochondrial elongation were also observed in the neurons of these older mutant flies (S7 Fig). Note, however, that the changes in the actin cytoskeleton and mitochondria were significantly less in magnitude than those present in tau transgenic flies (compare S7D Fig and Fig 2D; S7F Fig and Fig 3B), consistent with a lack of overt neurodegeneration in the Lrrk mutant flies (Fig 1).

## Discussion

While Parkinson disease and the tauopathies, including Alzheimer disease, share some clinical and pathological similarities, the disorders have generally been seen as distinct entities. However, the observation of tau aggregation and deposition as the primary neuropathology in some Parkinson disease patients with LRRK2 mutations and the implication of tau in sporadic Parkinson disease patients through genome-wide association studies has raised the intriguing possibility that the disorders share a common pathogenesis. Here, we describe a plausible molecular basis for the interaction between Lrrk and tau. We have previously demonstrated, using genetic and biochemical approaches, that tau exerts neurotoxicity by binding to and stabilizing F-actin [36], which promotes mislocalization of the critical mitochondrial fission protein Drp1 and subsequent mitochondrial dysfunction [18,57–61]. We now show that the single *Drosophila* LRRK family homolog Lrrk acts in the same pathway. Manipulating Lrrk expression in tau transgenic flies further increases F-actin levels, decreases Drp1 localization to mitochondria, enhances mitochondrial dysfunction, and promotes neurodegeneration. Fibroblasts from patients with LRRK2-GS mutations show increased numbers of F-actin bundles [21], consistent with our evidence for abnormal stabilization of F-actin following Lrrk manipulation (Fig 2A, 2C and 2D).

To probe the molecular mechanisms underlying in vivo stabilization of F-actin in Lrrk mutant animals, we performed a series of biochemical studies with purified LRRK2 and actin. Addition of LRRK2 to purified actin in vitro has previously been associated with a shift in actin from F-actin to G-actin, as monitored by cosedimentation [19]. Consistent with these findings, we demonstrate here that LRRK2 promotes depolymerization of actin (Fig 6A–6D) by directly severing actin filaments (Fig 6G–6J). Importantly, severing activity appears preferentially associated with the monomeric state of LRRK2, while oligomerization is correlated with loss of LRRK2-mediated severing. Further, we find that disease-associated LRRK2 has an increased propensity to oligomerize with wild-type LRRK2 and, under these conditions, reduced ability to sever actin (Fig 6I and 6J).

These biochemical findings have implications for the mechanism of LRRK2 action in disease. Both loss of function [62–64] and gain of function [65] mechanisms have previously been proposed in LRRK2-associated Parkinson disease. Our biochemical data suggest that mutant LRRK2 may have a dominant negative effect. These data fit well with our genetic findings that loss of Lrrk function using either a genetic mutation or transgenic RNAi strongly enhances the toxicity of human tau (Fig 1A). Further, our biochemical data demonstrating that under conditions favoring oligomerization of wild-type LRRK2 actin-severing activity is decreased fits with our observation that increasing expression of wild-type Lrrk enhances tau neurotoxicity (Fig 1A). We thus propose that at lower levels, Lrrk acts to sever actin and maintain normal actin dynamics and downstream mitochondrial dynamics. Disease-associated mutations in LRRK2 promote oligomerization of the protein, reduce actin-severing activity, and result in overstabilization of the actin cytoskeleton (Fig 6N). In the case of the disease-associated mutation G2019S, increased kinase activity may favor formation of dimeric LRRK2 [66], with a resultant decrease in active, actin-severing monomeric LRRK2 (Fig 6, S1 Fig). However, we note that expression of a form of Lrrk with mutations in three residues key for kinase activity can still promote tau toxicity to a limited extent (S1 Fig). Thus, Lrrk kinase activity may not be absolutely required for toxicity, or there may be multiple pathways mediating Lrrk toxicity. These findings are consistent with a potential therapeutic benefit to LRRK2 kinase inhibition [67], particularly in the context of increased LRRK2 expression or activity.

The dosage sensitivity to wild-type Lrrk that we demonstrate in vivo (Fig 1) and model biochemically (Fig 6) is consistent with recovery of noncoding Parkinson disease risk variants at the LRRK2 locus, which presumably predispose to disease by modulating LRRK2 expression [11]. Indeed, elevated levels of LRRK2 have been reported in patients with Parkinson disease [68,69]. Our findings are also consistent with prior cell culture data demonstrating a strong dosage dependence of LRRK2 toxicity [70]. However, we cannot exclude the possibility that loss of Lrrk function enhances tau neurotoxicity through a different mechanism than overexpression of Lrrk. Indeed, depolymerization [71] as well as excess stabilization of actin inhibits Drp1 localization and disrupts mitochondrial dynamics, consistent with a requirement for proper actin dynamics in localizing Drp1 to mitochondria. Thus, increasing expression of an actin-severing protein (Fig 6) might disrupt mitochondrial Drp1 localization (Figs 3 and 5) by destabilizing actin. However, our data demonstrating increased F-actin and numbers of actin-rich rods when Lrrk is overexpressed argues against this possibility (Fig 2), and we thus rather favor a dominant negative mechanism for enhancement of tau neurotoxicity by Lrrk manipulation in neurons (Fig 6). Nonetheless, we note that knockout of LRRK2 is associated with lung and kidney pathology not seen in animals with knockin of Parkinson disease—associated LRRK2 mutations [72,73]. Similarly, while actin dynamics in myeloid cells is altered in a Wiskott—Aldrich syndrome protein family member 2 (WAVE2)-dependent fashion in both LRR2 knockout and LRRK-G2019S knockin mice, F-actin levels are decreased in knockout microglia and increased in knockin microglia [25]. Further work will be required to investigate fully the mechanisms underlying LRRK2-mediated dysfunction in various tissues and cell types and their contribution to human disease phenotypes.

The multiple, seemingly disparate cellular pathways influenced by LRRK2 in various experimental organisms and systems has been a puzzling aspect of LRRK2 biology [11,74–76]. Our data suggest a possible explanation for these diverse results. A primary effect of LRRK on actin stabilization in disease states is consistent with the cellular pathologies previously linked to LRRK dysfunction, including altered vesicle trafficking [72,77–83], miRNA and translational regulation [32,84], and nucleoskeletal changes [85], because these processes are all regulated by the actin cytoskeleton [46,86–89]. Alternatively, LRRK2 could promote disease pathogenesis through multiple pathways, including by influencing phosphorylation and transmission [48,90–92] of tau.

Consistent with our pharmacological rescue of tau neurotoxicity and mitochondrial dysfunction in vivo (Fig 4), chemical depolymerization of actin with latrunculin A reverses actin cytoskeletal abnormalities in patient-derived cells [21]. Manipulation of the actin cytoskeleton may thus represent a new target for therapy development in Parkinson disease as well as in other parkinsonian disorders and in the larger group of tauopathies. Although manipulation of the microtubule cytoskeleton for therapeutic purposes has a long history and multiple effective agents in clinical use for diverse disorders ranging from cancer to gout [93], targeting of the actin cytoskeleton represents a relatively unexplored therapeutic arena [47]. While caution certainly is warranted given the essential roles of the actin cytoskeleton in normal biology, manipulation of actin polymerization by targeting actin-binding proteins has shown promise in preclinical studies in cancer [94] and renal disease [95]. Similarly, in the current studies, we observed rescue of mitochondrial functional defects and neurotoxicity without excessive toxicity following systemic delivery of the pharmacological agents latrunculin A or the cytochalasins (Fig 4). These findings complement our prior work demonstrating that genetic destabilization of the actin cytoskeleton is protective in tauopathies [18,46].

The mitochondrial pathology we observe is consistent with prior reports of elongated, fusiform mitochondria in LRRK2 G2019S knockin mice [96]. In contrast, a separate study did not observe enhanced neuropathology in mice expressing P301S mutant human tau under the control of the mouse prion promoter and coexpressing mutant human LRRK2 R1441G from a bacterial artificial chromosome [97]. The reasons for the differences between our study and the work of Mikhail and colleagues [97] are not clear; however, the divergent results may reflect the forms of tau and LRRK2 expressed and levels and cellular expression patterns of the transgenes. Additional genetic analyses in LRRK2 knockin and knockout rodent models with concomitant expression of wild-type and disease-linked mutant versions of human tau may provide important information regarding the in vivo interactions of these two proteins. Knockout of both LRRK1 and LRRK2 in the brain may be required in these studies [64].

Our current findings are particularly intriguing in the context of Parkinson disease because two other proteins implicated in familial Parkinson disease, parkin and phosphatase and tensin homolog induced putative kinase 1 (PINK1), play important roles in mitochondrial dynamics and mitophagy [11,27,98]. Of note, these recessive disorders fit well within the category of parkinsonian neurodegeneration because they have clinical features atypical for sporadic Parkinson disease and are only infrequently characterized by Lewy body formation [7,9]. Thus, interference with the molecular machinery controlling mitochondrial dynamics, quality control, and function, rather than a specific neuropathology, may best define the clinicopathological entity that is parkinsonian neurodegeneration.

In summary, we have outlined here a molecular pathway that plausibly connects the pathogenesis of the two most common, seemingly disparate neurodegenerative disorders: Parkinson disease and the tauopathies. Our findings correlate with prior implication of mitochondrial dysfunction in recessive forms of Parkinson disease and have important implications for therapy. Our data raise the possibility of a dominant negative, loss-of-function mechanism in LRRK2-related Parkinson disease and thus suggest caution when considering strong or complete inhibition of LRRK2 in therapeutic approaches to the disorder. Therapies that target excess stabilization of F-actin (Fig 4) or mitochondrial dysfunction may be promising avenues for further investigation.

## Materials and methods

### Ethics statement

Mice were housed and treated in accordance with the National Institutes of Health (NIH) Guide for the Care and Use of Laboratory Animals. All animal procedures were approved and performed in accordance with the Mayo Clinic Institutional Animal Care and Use committee and the University of Florida Institutional Animal Care and Use committee. Mice were maintained in a pathogen-free facility on a 12-hour light/dark cycle with water and food provided ad libitum. All mice were euthanized by cervical dislocation to maintain the brain biochemistry by avoiding anesthesia-induced tau changes.

### Genetics

All *Drosophila* crosses and aging were performed at 25 °C. Assays were performed on 10-day-old flies, with the exception of experiments in Fig 6, S5 and S6 Figs (1–3 days of age) and S7 Fig (20 days of age). The panneuronal driver *elav-GAL4* was used for all experiments. The human *UAS-tau*^*R406W*^ line has been described previously [27]. The following *Drosophila* stocks were kindly provided by the indicated investigators: *Lrrke*^*03680*^, *UAS-Lrrk RNAi*, *UAS-Lrrk*^*wt*^, *UAS-Lrrk*^*I1915T*^, and *UAS-Lrrk*^*Y1383C*^ by Dr. Bingwei Lu; *UAS-Lrrk*^*G1914S*^ by Dr. Ming Guo; *Lrrk*^*HA*^ knockin animals by Dr. Patrick Verstreken; *UAS-CD8-PARP-Venus* transgenic caspase reporter by Dr. Darren Williams; *FLAG-FlAsH-HA-Drp1* by Dr. Hugo Bellen; *UAS-mito-GFP* by Dr. Thomas Schwarz; and *UAS-SCA3(MJD)-78* by Dr. Nancy Bonini. *elav-GAL4*, *UAS-EGFP*, and *UAS-MitoTimer* were obtained from the Bloomington *Drosophila* Stock Center. All mouse experiments for the tau transgenic strains rTg4510 and rTg4510-LRRK2-WT BAC [48] were performed at 5.5 months of age. Controls were age- and gender-matched nontransgenic animals. Mice were housed and treated in accordance with the NIH Guide for the Care and Use of Laboratory Animals. All animal procedures were approved and performed in accordance with the Mayo Clinic Institutional Animal Care and Use committee and the University of Florida Institutional Animal Care and Use committee. Mice were maintained in a pathogen-free facility on a 12-hour light/dark cycle with water and food provided ad libitum.

The parental Tau^P301L^ responder line and parental tTA activator line were generated and maintained on an FVB and 129/S6 background, respectively. The parental bacterial artificial chromosome (BAC)-LRRK2 mice were maintained on an FVB background. Mice from the Tau^P301L^ responder line were crossed with mice from the BAC-LRRK2 mouse line for one generation to obtain LRRK2. Tau^P301L^ responder mice on an FVB background. LRRK2. Tau^P301L^ responder mice were then crossed with mice from the tTA activator line to obtain the resultant F1 LRRK2/Tau^P301L^ mice on a 50% FVB, 50% 129S background [48]. For all *Drosophila* and mouse experiments, equivalent numbers of male and female animals were used.

### Antibodies

A polyclonal antibody against Lrrk was generated in rabbit using Lrrk 1–336 amino acids as the epitope using the services of Covance. This antibody was used at a dilution of 1:1000 in 5% BSA for western blotting. The following antibodies were used at the indicated concentrations: mouse monoclonal actin (JLA20), 1:500, Developmental Studies Hybridoma Bank; rabbit polyclonal actin (A2066), 1:1000, Sigma; HA-11, 1:200, Covance; GAPDH, 1:200,000, Abcam; tau (tau5), 1:20,000, Developmental Studies Hybridoma Bank; elav, 1:50, Developmental Studies Hybridoma Bank; cleaved PARP (E51), 1:200,000, Abcam; Drp1, 1:200, SantaCruz Biotechnology; GFP (N86/8), 1:1000, NeuroMab; ATPVa, 1:500, Novex; and TH, 1:200, Immunostar.

### Gel electrophoresis and immunoblotting

*Drosophila* brains were homogenized in 2X sample buffer and analyzed by 4%–12% SDS-PAGE and immunoblotted according to standard protocols. Each blot was repeated at least three times with similar results and quantified using ImageJ. An image of a representative blot is shown in the figures. For native PAGE and silver staining, recombinant wild-type human LRRK2 or G2019S mutant human LRRK2 protein samples (5 nM) were prepared in 2X native gel sample buffer (62.5 mM Tris-HCL, pH 6.8, 25% glycerol, and 1% bromophenol blue). The samples were run on 4%–20% prescast tris gels (Lonza 58517) in running buffer without SDS (25 mM Tris, 192 mM Glycine). Silver staining was performed on the gel using the Thermo Scientific Pierce Silver Stain Kit (Cat. #24162) following the manufacturer’s protocol.

### Immunostaining and image analysis

Quantification of the number of neurons with caspase activation and PCNA staining was performed on 4 μm paraffin-embedded tissue sections, and positive cells throughout the entire brain were counted. Mitochondrial length was assessed by imaging *Drosophila* Kenyon neurons and murine hippocampal pyramidal neurons using laser scanning confocal microscope. Individual mitochondrial length was measured by freehand line length using ImageJ (http://rsbweb.nih.gov/ij). All the mitochondria quantified for length were also scored for colocalization. Mitochondrial interconnectivity was assessed using the ImageJ macro “mitochondrial morphology” publicly available at http://imagejdocu.tudor.lu/doku.php. The average area/perimeter normalized to the average circularity was taken as the measure of mitochondrial interconnectivity. Pearson’s coefficient was calculated by circling individual cells and using the ImageJ macro Coloc2. Fifty cells were analyzed for each genotype for Pearson’s coefficient calculations. Percent colocalization of Lrrk and F-actin or mitochondria was also assessed using Coloc2. The number of actin-rich rods was determined by counting all rod-shaped or round structures over 3 microns in size that stained for actin. Actin rods were counted throughout the entire *Drosophila* brain. All the actin rods experiments in mice were done blind, and the counts were performed without the knowledge of the genotypes. For assessment of fluorescence in *Drosophila* brains, the samples were processed simultaneously using the same acquisition parameters. For the quantification of fluorescence, average pixel density from two-dimensional projections of z-stacks for the entire brain was computed using ImageJ. The density of Kenyon cells per μm^2^ was determined on H&E stained paraffin sections. The density of TH-positive cells per μm^2^ was determined on paraffin sections of the anterior medulla, as described [39].

### Stress assays

#### MitoSOX

The production of superoxide in the mitochondria was assessed using the MitoSOX Red Reagent (Molecular Probes). Fresh fly brains were dissected in 5 μM MitoSOX reagent. The brains were placed in 50 μl of the reagent and incubated at 37 °C for 30 minutes. After three 5-minute washes in PBS at 37 °C, the brains were fixed in 4% paraformaldehyde for 20 minutes and mounted on glass slides. Confocal images of the brains were taken using the same settings for control and experimental samples. For the quantification of fluorescence, average pixel intensity of two-dimensional projections of confocal z-stacks for the entire brain was measured using the ImageJ software.

#### MitoTimer

Fresh brains were dissected from either control or tau transgenic flies expressing MitoTimer. Two brains of each genotype were placed in each well of a 384-well black clear-bottom plate containing 20 μl PBS. The red fluorescence was measured at the excitation/emission wavelengths 543/572 and the green fluorescence at 488/518. The ratio of the red over green fluorescence was taken as an indicator of the level of reporter oxidation.

#### ROSstar

The ROSstar 650 hydrocyanine probe (Li-Cor) was used to assess the levels of ROSs. Brains from control or tau transgenic flies were dissected in 200 μM ROSstar reagent and incubated in 50 μl reagent at 37 °C for 20 minutes. After three 5-minute washes in PBS at 37 °C, two brains each were placed in wells containing 20 μl PBS, and the fluorescence was measured at the excitation/emission wavelengths of 640/660.

### ELISA

Freshly dissected brains from 10-day-old flies were homogenized in 50 μl actin stabilization buffer from the G-actin/F-actin in vivo assay kit from Cytoskeleton Inc. (Cat. #BK037). Ten μl of the homogenate was used in the F-actin ELISA assay from MyBioSource Inc. (Cat. #MBS702018), and the assay was performed according to the manufacturer’s protocol. The remainder of the homogenate was used in western blotting to ensure equal levels of total actin and the input. Each experiment was performed with two technical replicates.

### Drug treatments

Flies were collected on the day of eclosion and were fed 0, 5, or 10 μM of drug dissolved in ethanol and mixed into *Drosophila* culture medium, as described [99,100]. In each vial, 10–12 flies were kept, and the vials were changed every third day and analyzed at 10 days.

### Murine neuronal counts

Sagittal brain sections were stained with H&E to visualize structure and align slides. Matching brain sections for each animal were scanned into the ScanScope XT scanner and visualized through ImageScope version 11.2.0.780 software (Aperio). An individual (MJH) who was blinded to genotype and sex of animals outlined the regions of interest. He then performed a direct count of all cells within the CA1, placing a mark on each cell that was counted. If the matched section was ripped within the region to be counted, that animal was excluded from analysis.

### Actin depolymerization assay

Actin depolymerization assays were performed using the fluorescent form of the Actin Polymerization Biochem Kit from Cytoskeleton Inc. (Cat. #BK003), as described by the manufacturer. Pyrene-labeled actin was polymerized at room temperature for 1 hour in actin polymerization buffer (containing 2 mM MgCl_2_ and 2 mM ATP) and then mixed either with buffer or with human recombinant LRRK2, LRRK2-G2019S, or a 1:1 mixture of the two. Actin was used at a final concentration of 4 μM and the LRRK2 proteins at 1 nM. The samples were read every 30 seconds in a plate reader at the excitation/emission wavelengths of 350/410 for 1 hour. The experiment was repeated three times with two technical replicate each time per sample. The values were normalized to 0 at the starting time. Each data point represents the mean of three separate experiments.

### Actin severing assay

Actin (4 μM) was polymerized in actin polymerization buffer (containing 2 mM MgCl_2_ and 2 mM ATP) at room temperature for 1 hour. Polymerized actin was incubated with LRRK2, LRRK2-GS, or mix (1 nM final concentration) for 2 minutes. Fluorescently labeled phalloidin (Acti-stain 555) was added to a final concentration of 2 μM, and the samples were diluted 50-fold with PBS. Of each sample, 2 μl was adsorbed on coverslips coated with 0.01% poly-L-lysine and imaged using a fluorescence microscope. The filament lengths were quantified using ImageJ software by freehand drawing tool. Three different randomly selected areas were quantified for each sample, with at least 10 filaments measured per area. The experiment was repeated three times.

### Phalloidin staining

Brains from 20-day-old flies were dissected in PBS and fixed in 4% PFA on ice for 30 minutes. After a 10-minute incubation in 0.3% Triton-X, the brains were stained with Acti-stain 555 phalloidin (Cytoskeleton Inc., Cat. #PHDH1) at a concentration of 14 nM for 30 minutes in the dark. Brains were then washed three times in PBS for 60 minutes each, mounted, and imaged using confocal microscopy.

### Phalloidin precipitation

Forty fly heads were homogenized and centrifuged at 800 x g to pellet debris. The supernatant was incubated with 0.3 units biotinylated phalloidin (Molecular Probes), followed by precipitation with streptavidin-coupled Dynabeads (Invitrogen). Flies were 10 days old in all coprecipitations.

### Statistical analysis

All reported *n* values are biological replicates. The sample sizes used were similar to the ones reported in previous publications [18,28]. For *Drosophila* immunostaining and genetic reporter- and dye-labeling experiments, the sample size was six per genotype and time point. Western blot quantifications were performed on at least three independent blots. The ELISA experiment was repeated three times, with two technical replicates per sample. The sample sizes for mouse experiments were determined by power analyses. For the histological studies, we determined that a sample size of five is sufficient to detect 20% difference between control and experimental genotypes with a power of 80%. Exact sample size for each experiment is provided in the figure legends. Data collection and analysis in mouse experiments were performed blinded to the conditions of the experiment. Experiments in flies were not performed blinded. Statistical analysis was performed using one-way ANOVA, and multiple comparisons among the data sets were performed. Variance was similar between groups compared.

## Supporting information

S1 Fig (A) Increasing or decreasing *Drosophila* Lrrk does not alter the toxicity of mutant SCA3, as measured by counting the number of Kenyon neurons, presented as the number of neurons per μm^2^. Control is *elav-GAL4/+*. (B, C) Expression of Lrrk-3KD has reduced ability to enhance tau neurotoxicity, as monitored by caspase activation (B) or reactivation of the cell cycle as assessed by immunostaining for PCNA (C). *n* = 6 per genotype. *P < 0.01, ANOVA with supplementary Neuman—Keuls. Control is *elav-GAL4/*+; *UAS-CD8-PARP-Venus/+* in B and *elav-GAL4/+* in A, C. Flies are 10 days old. See S1 Data for individual numerical values underlying the summary data displayed in A—C. Lrrk, leucine-rich repeat kinase; Lrrk-3KD; ns, not significant; PCNA, proliferating cell nuclear antigen.
(TIF)

S2 Fig Effects of expressing mutant Lrrk or wild-type human LRRK2. (A) Western blot showing that Lrrk-GS levels are similar to endogenous Lrrk levels when expressed in *Lrrk* mutant background. (B) Expression of Lrrk-GS in tau transgenic flies in the *Lrrk* mutant background does not significantly increase the number of neurons with activated caspase, indicating no increase of neuronal toxicity. (C) There is no significant increase in cell cycle activation in postmitotic neurons when Lrrk-GS is expressed with tau in flies in the *Lrrk* mutant background. (D) There is no significant increase in the number of actin rods when Lrrk-GS is expressed with tau in flies with *Lrrk* mutant background. (E—G) Expression of human LRRK2 enhances tau neurotoxicity, as observed by caspase activation (E) and by cell cycle activation in postmitotic neurons (F). (G) The number of actin rods in the brains of tau transgenic flies is increased in the presence of human LRRK2. *n* = 6 per genotype. *P < 0.01, ANOVA with supplementary Neuman—Keuls. Control is *elav-GAL4/+* in A, B, D, F, and G and *elav-GAL4/*; *UAS-CD8-PARP-Venus/+* in C, E. Flies are 10 days old. See S1 Data for individual numerical values underlying the summary data displayed in B—G. LRRK2, leucine-rich repeat kinase 2; Lrrk-GS, Lrrk carrying the G1914S mutation.
(TIF)

S3 Fig Increasing or decreasing Lrrk enhances human tau—induced loss of dopaminergic neurons. (A) Representative images showing TH-positive neurons in the anterior medulla of the flies of the indicated genotypes. (B) Quantification of TH-positive neuron loss with tau expression, which is enhanced by altering Lrrk expression. *n* = 6 per genotype. *P < 0.01, ANOVA with supplementary Neuman—Keuls. Control is *elav-GAL4/+*. Flies are 10 days old. See S1 Data for individual numerical values underlying the summary data displayed in B. Lrrk, leucine-rich repeat kinase; TH, tyrosine hydroxylase.
(TIF)

S4 Fig Rescue of Lrrk-modified tau neurotoxicity by actin-destabilizing drugs. (A) Improvement in tau and Lrrk neurotoxicity with feeding of the actin-depolymerizing compounds LatA, Cyto-B, or Cyto-D, as assessed by the number activated caspase-positive cells. (B) Cell cycle activation is reduced in flies treated with LatA, Cyto-B, or Cyto-D. All drugs were used at 25 μM. *n* = 6 per genotype and treatment. *P < 0.01, ANOVA with supplementary Neuman—Keuls. Control is *elav-GAL4/+*; *UAS-CD8-PARP-Venus/+* in A and *elav-GAL4/+* in B. Flies are 10 days old. See S1 Data for individual numerical values underlying the summary data displayed in A, B. Cyto-B, cytochalasin B; Cyto-D, cytochalasin D; LatA, latrunculin A; Lrrk, leucine-rich repeat kinase.
(TIF)

S5 Fig Mitochondrial morphology in tau transgenic mice and Lrrk oligomerization in vivo. (A) Higher magnification views of immunofluorescent images of mouse brain sections stained with NeuN to visualize the hippocampal pyramidal neurons and ATPVa to demonstrate mitochondrial morphology show elongation in tau transgenic mice. Scale bar represents 5 μm. Mice are 5.5 months old. (B) Native gel showing enhanced dimerization and oligomerization in flies overexpressing wild-type Lrrk or expressing mutant Lrrk-GS. Control is *elav-GAL4/*+; *Lrrk*^*HA*^/+. (C) Quantification of the oligomerization of Lrrk from three different blots. *P < 0.01, ANOVA with supplementary Neuman—Keuls. Flies are 10 days old. See S1 Data for individual numerical values underlying the summary data displayed in C. ATPVa, vacuolar protein-ATPase A-subunit; HA, hemagglutinin; Lrrk, leucine-rich repeat kinase; Lrrk-GS, Lrrk carrying the G1914S mutation; NeuN, neuronal nuclei.
(TIF)

S6 Fig Lrrk colocalizes with mitochondria. (A, B) Colocalization of *Drosophila* Lrrk, visualized with an HA antibody, mitochondria in flies expressing HA-tagged Lrrk from its endogenous promoter, and mito-GFP (arrows). *n* = 3. Genotype: *elav-GAL4/+*; *UAS-mito-GFP*/+; *Lrrk*^*HA*^/+. (C) Images of control flies (not expressing HA-tagged Lrrk) stained using the HA antibody, demonstrating no significant nonspecific immunoreactivity. Genotype: *elav-GAL4/+*; *UAS-mito-GFP*/+. Scale bars represent 5 μm. See S1 Data for individual numerical values underlying the summary data displayed in B. HA, hemagglutinin; Lrrk, leucine-rich repeat kinase; mito-GFP, mitochondrially directed GFP.
(TIFF)

S7 Fig Excess stabilization of actin in aged Lrrk mutant flies. (A) Phalloidin precipitation of F-actin showing increased actin stabilization in flies with loss of Lrrk (*Lrrke*^*03680*^) at 20 days of posteclosion age. Quantification from three separate blots is shown in the lower panel. (B) Representative images of freshly dissected *Drosophila* brains stained with fluorescent phalloidin. (C) Quantification of the fluorescence intensity of the entire fly brain showing enhanced actin stabilization with loss of Lrrk. (D) Quantification of the number of actin rods in the brains of either control or flies with loss of Lrrk. (E) Quantification of the number of mitochondria colocalized with Drp1 shows reduced mitochondrial localization of Drp1 in flies with reduced Lrrk. (F) Quantification of mitochondrial length shows elongated mitochondria in flies with reduced levels of Lrrk. *n* = 6 per genotype (B-F). *P < 0.05, t-test. Control is *elav-GAL4/+* in A, B, C, and D and *elav-GAL4/+*; *UAS-mito-GFP/+*; *HA-Drp1/+* in E, F. Flies are 20 days old. See S1 Data for individual numerical values underlying the summary data displayed in A, C—F. Drp1, dynamin-1-like protein; F-actin, filamentous actin; HA, hemagglutinin; Lrrk, leucine-rich repeat kinase; mito-GFP, mitochondrially directed GFP.
(TIF)

S1 Data Individual numerical values, which underlie the summary data displayed in the following figure panels: Figs 1A, 1C, 1D, 1E, 2A, 2B, 2D–2H, 3B–3F, 3H–3J, 4A–4F, 4H, 4J, 5C, 5D, 5G, 5H, 5I, 6A–6D, 6F, 6H, 6J and 6M; S1A–S1C, S2B–S2G, S3B, S4A, S4B, S5C, S6B, S7A and S7C–S7F. (DOCX)

## Abbreviations

BAC: bacterial artificial chromosome
CA1: cornu ammonis 1
CAMKIIα-tTA: calcium/calmodulin-dependent protein kinase II reverse transactivator
Drp1: dynamin-1-like protein
elav: embryonic lethal abnormal vision
ELISA: enzyme-linked immunosorbent assays
F-actin: filamentous actin
FTDP-17: frontotemporal dementia with parkinsonism linked to Chromosome 17
GFP: green fluorescent protein
H&E: hematoxylin and eosin
HA: hemagglutinin
LRRK: leucine-rich repeat kinase
LRRK2-GS: LRRK2 with the G2019S mutation
mito-GFP: mitochondrially directed GFP
NICHD: National Institute of Child Health and Human Development
NIH: National Institutes of Health
PAGE: polyacrylamide gel electrophoresis
PCNA: proliferating cell nuclear antigen
PINK1: phosphatase and tensin homolog induced putative kinase 1
ROS: reactive oxygen species
SCA3: spinocerebellar ataxia type 3
TH: tyrosine hydroxylase
UAS: upstream activating sequence
WAVE2: Wiskott—Aldrich syndrome protein family member 2
