# Supplementary figures and images for: Lrrk promotes tau neurotoxicity through dysregulation of actin and mitochondrial dynamics
Source: PLoS Biol. 2018 Dec 20;16(12):e2006265. doi: 10.1371/journal.pbio.2006265 (PMC6319772; doi:10.1371/journal.pbio.2006265)

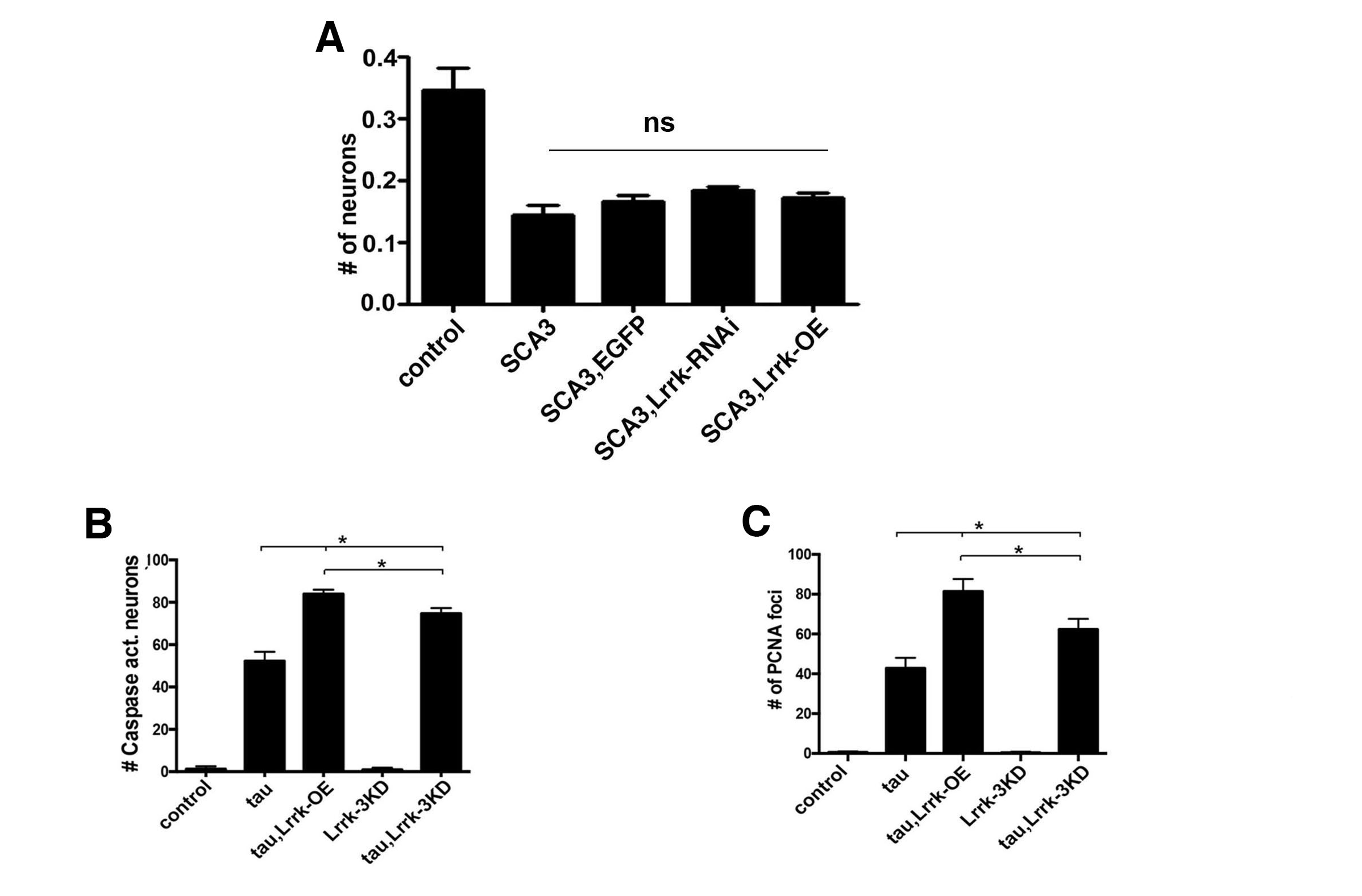

Supplement: S1 Fig — (A) Increasing or decreasing Drosophila Lrrk does not alter the toxicity of mutant SCA3, as measured by counting the number of Kenyon neurons, presented as the number of neurons per μm2. Control is elav-GAL4/+. (B, C) Expression of Lrrk-3KD has reduced ability to enhance tau neurotoxicity, as monitored by caspase activation (B) or reactivation of the cell cycle as assessed by immunostaining for PCNA (C). n = 6 per genotype. *P < 0.01, ANOVA with supplementary Neuman—Keuls. Control is elav-GAL4/+; UAS-CD8-PARP-Venus/+ in B and elav-GAL4/+ in A, C. Flies are 10 days old. See S1 Data for individual numerical values underlying the summary data displayed in A—C. Lrrk, leucine-rich repeat kinase; Lrrk-3KD; ns, not significant; PCNA, proliferating cell nuclear antigen. (TIF) [file pbio.2006265.s001.tif]

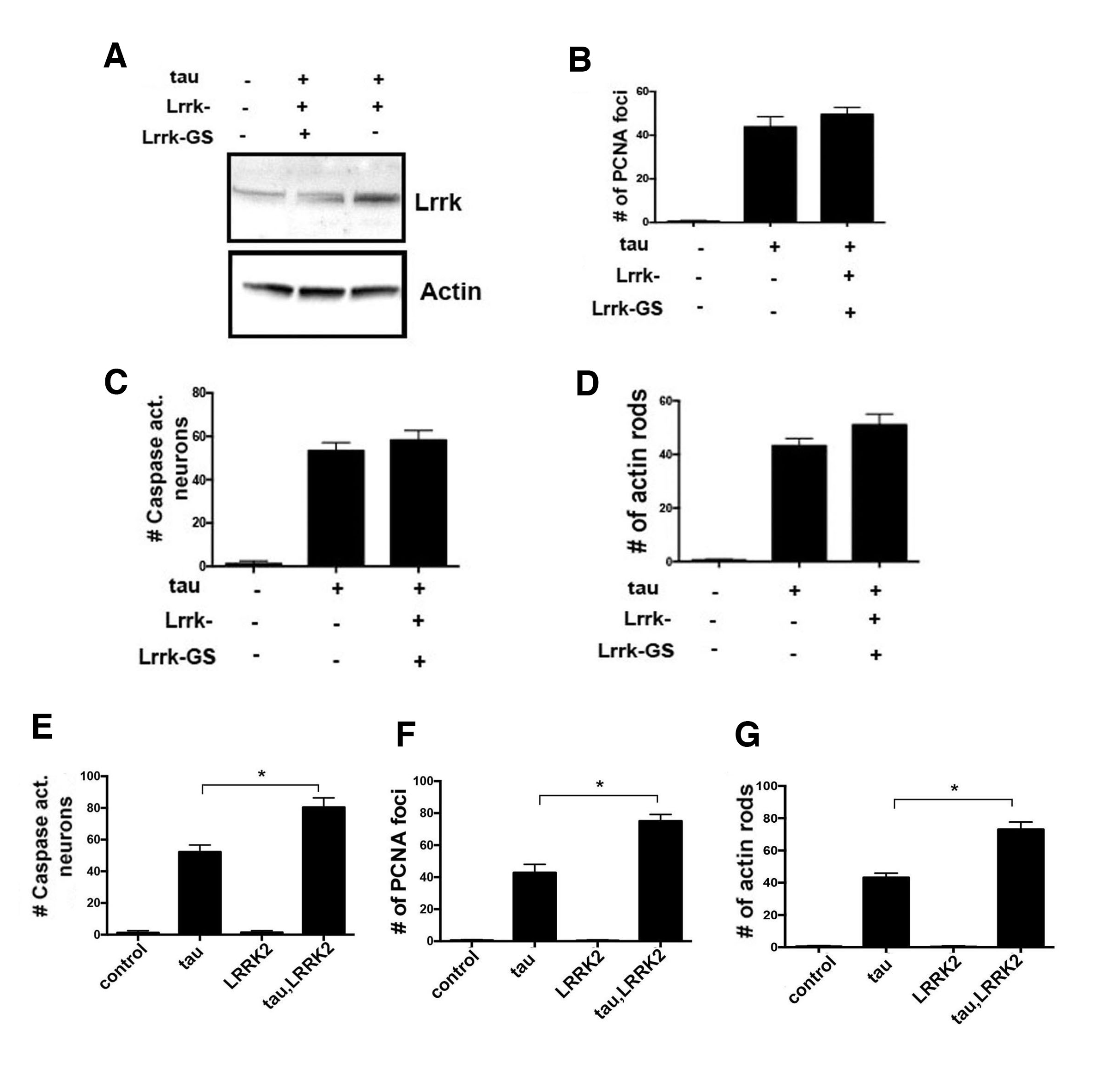

Supplement: S2 Fig — (A) Western blot showing that Lrrk-GS levels are similar to endogenous Lrrk levels when expressed in Lrrk mutant background. (B) Expression of Lrrk-GS in tau transgenic flies in the Lrrk mutant background does not significantly increase the number of neurons with activated caspase, indicating no increase of neuronal toxicity. (C) There is no significant increase in cell cycle activation in postmitotic neurons when Lrrk-GS is expressed with tau in flies in the Lrrk mutant background. (D) There is no significant increase in the number of actin rods when Lrrk-GS is expressed with tau in flies with Lrrk mutant background. (E—G) Expression of human LRRK2 enhances tau neurotoxicity, as observed by caspase activation (E) and by cell cycle activation in postmitotic neurons (F). (G) The number of actin rods in the brains of tau transgenic flies is increased in the presence of human LRRK2. n = 6 per genotype. *P < 0.01, ANOVA with supplementary Neuman—Keuls. Control is elav-GAL4/+ in A, B, D, F, and G and elav-GAL4/; UAS-CD8-PARP-Venus/+ in C, E. Flies are 10 days old. See S1 Data for individual numerical values underlying the summary data displayed in B—G. LRRK2, leucine-rich repeat kinase 2; Lrrk-GS, Lrrk carrying the G1914S mutation. (TIF) [file pbio.2006265.s002.tif]

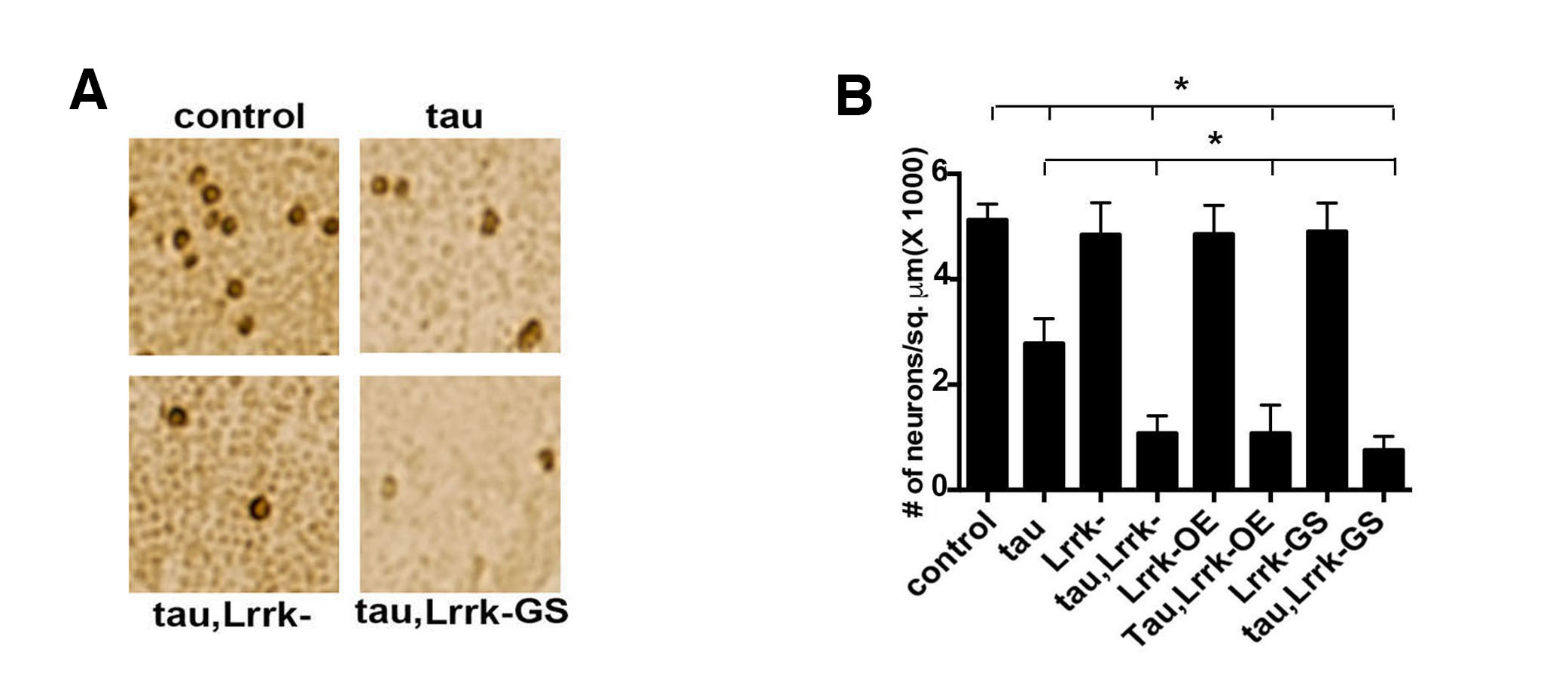

Supplement: S3 Fig — (A) Representative images showing TH-positive neurons in the anterior medulla of the flies of the indicated genotypes. (B) Quantification of TH-positive neuron loss with tau expression, which is enhanced by altering Lrrk expression. n = 6 per genotype. *P < 0.01, ANOVA with supplementary Neuman—Keuls. Control is elav-GAL4/+. Flies are 10 days old. See S1 Data for individual numerical values underlying the summary data displayed in B. Lrrk, leucine-rich repeat kinase; TH, tyrosine hydroxylase. (TIF) [file pbio.2006265.s003.tif]

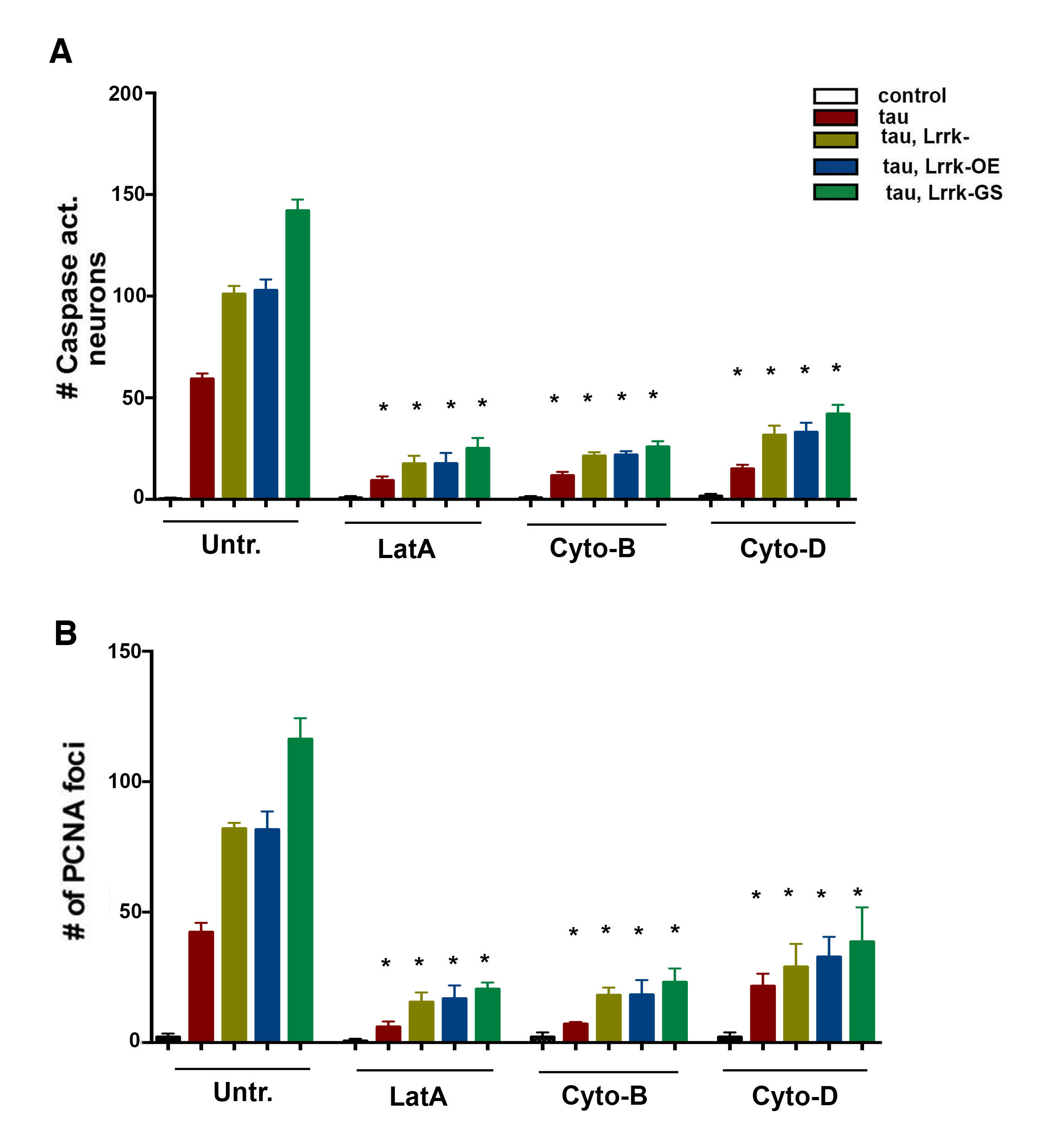

Supplement: S4 Fig — (A) Improvement in tau and Lrrk neurotoxicity with feeding of the actin-depolymerizing compounds LatA, Cyto-B, or Cyto-D, as assessed by the number activated caspase-positive cells. (B) Cell cycle activation is reduced in flies treated with LatA, Cyto-B, or Cyto-D. All drugs were used at 25 μM. n = 6 per genotype and treatment. *P < 0.01, ANOVA with supplementary Neuman—Keuls. Control is elav-GAL4/+; UAS-CD8-PARP-Venus/+ in A and elav-GAL4/+ in B. Flies are 10 days old. See S1 Data for individual numerical values underlying the summary data displayed in A, B. Cyto-B, cytochalasin B; Cyto-D, cytochalasin D; LatA, latrunculin A; Lrrk, leucine-rich repeat kinase. (TIF) [file pbio.2006265.s004.tif]

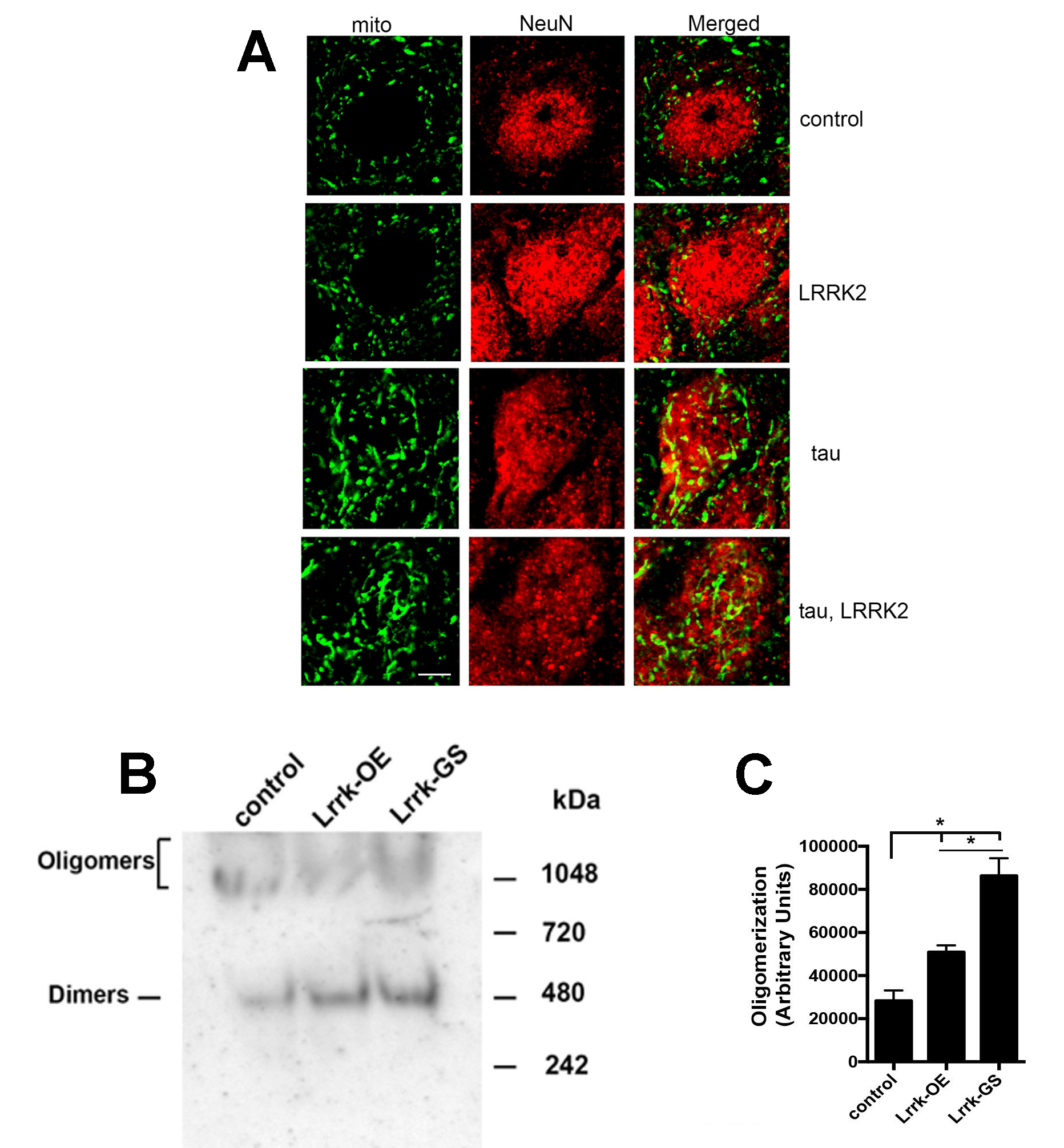

Supplement: S5 Fig — (A) Higher magnification views of immunofluorescent images of mouse brain sections stained with NeuN to visualize the hippocampal pyramidal neurons and ATPVa to demonstrate mitochondrial morphology show elongation in tau transgenic mice. Scale bar represents 5 μm. Mice are 5.5 months old. (B) Native gel showing enhanced dimerization and oligomerization in flies overexpressing wild-type Lrrk or expressing mutant Lrrk-GS. Control is elav-GAL4/+; LrrkHA/+. (C) Quantification of the oligomerization of Lrrk from three different blots. *P < 0.01, ANOVA with supplementary Neuman—Keuls. Flies are 10 days old. See S1 Data for individual numerical values underlying the summary data displayed in C. ATPVa, vacuolar protein-ATPase A-subunit; HA, hemagglutinin; Lrrk, leucine-rich repeat kinase; Lrrk-GS, Lrrk carrying the G1914S mutation; NeuN, neuronal nuclei. (TIF) [file pbio.2006265.s005.tif]

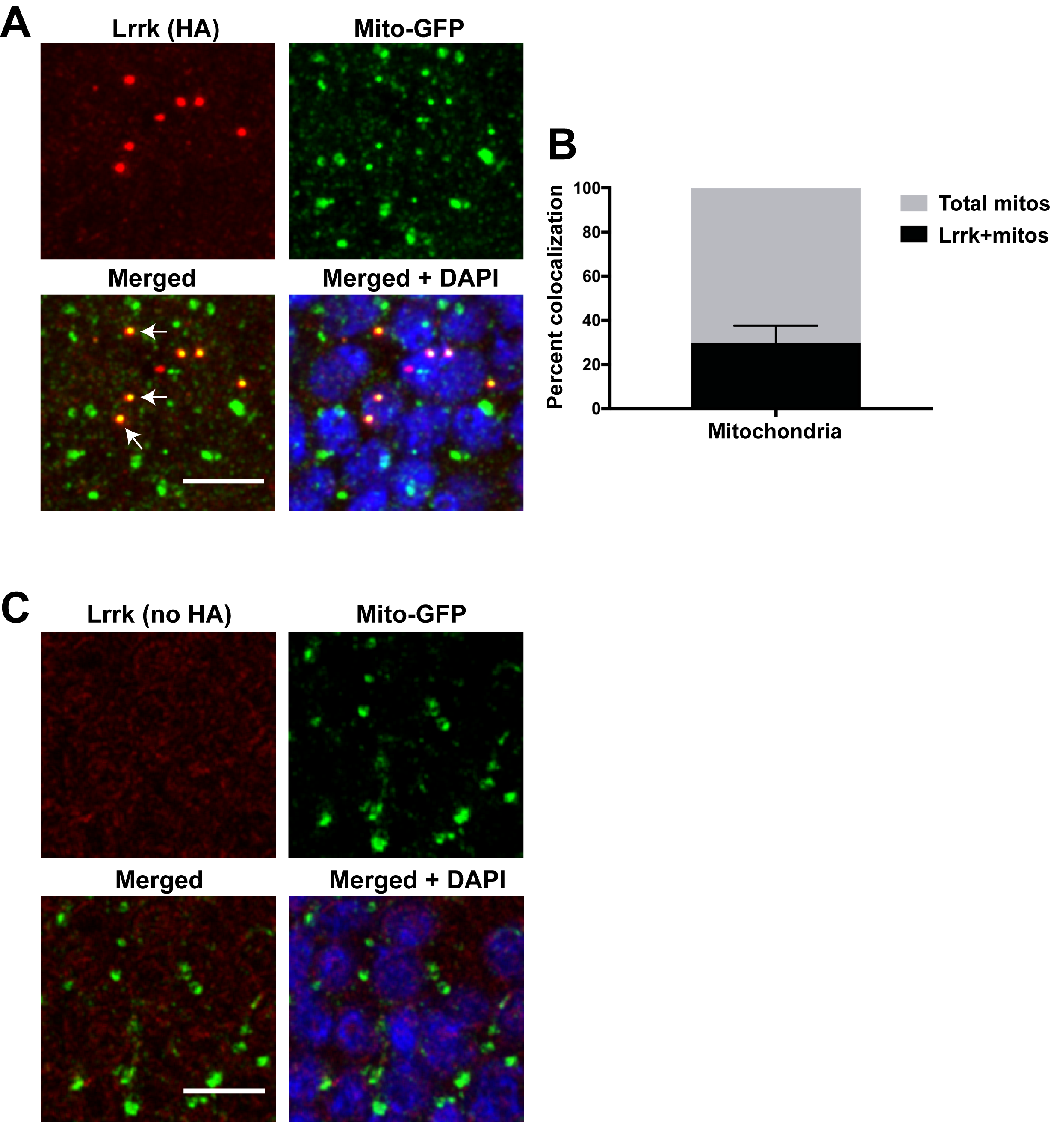

Supplement: S6 Fig — (A, B) Colocalization of Drosophila Lrrk, visualized with an HA antibody, mitochondria in flies expressing HA-tagged Lrrk from its endogenous promoter, and mito-GFP (arrows). n = 3. Genotype: elav-GAL4/+; UAS-mito-GFP/+; LrrkHA/+. (C) Images of control flies (not expressing HA-tagged Lrrk) stained using the HA antibody, demonstrating no significant nonspecific immunoreactivity. Genotype: elav-GAL4/+; UAS-mito-GFP/+. Scale bars represent 5 μm. See S1 Data for individual numerical values underlying the summary data displayed in B. HA, hemagglutinin; Lrrk, leucine-rich repeat kinase; mito-GFP, mitochondrially directed GFP. (TIFF) [file pbio.2006265.s006.tiff]

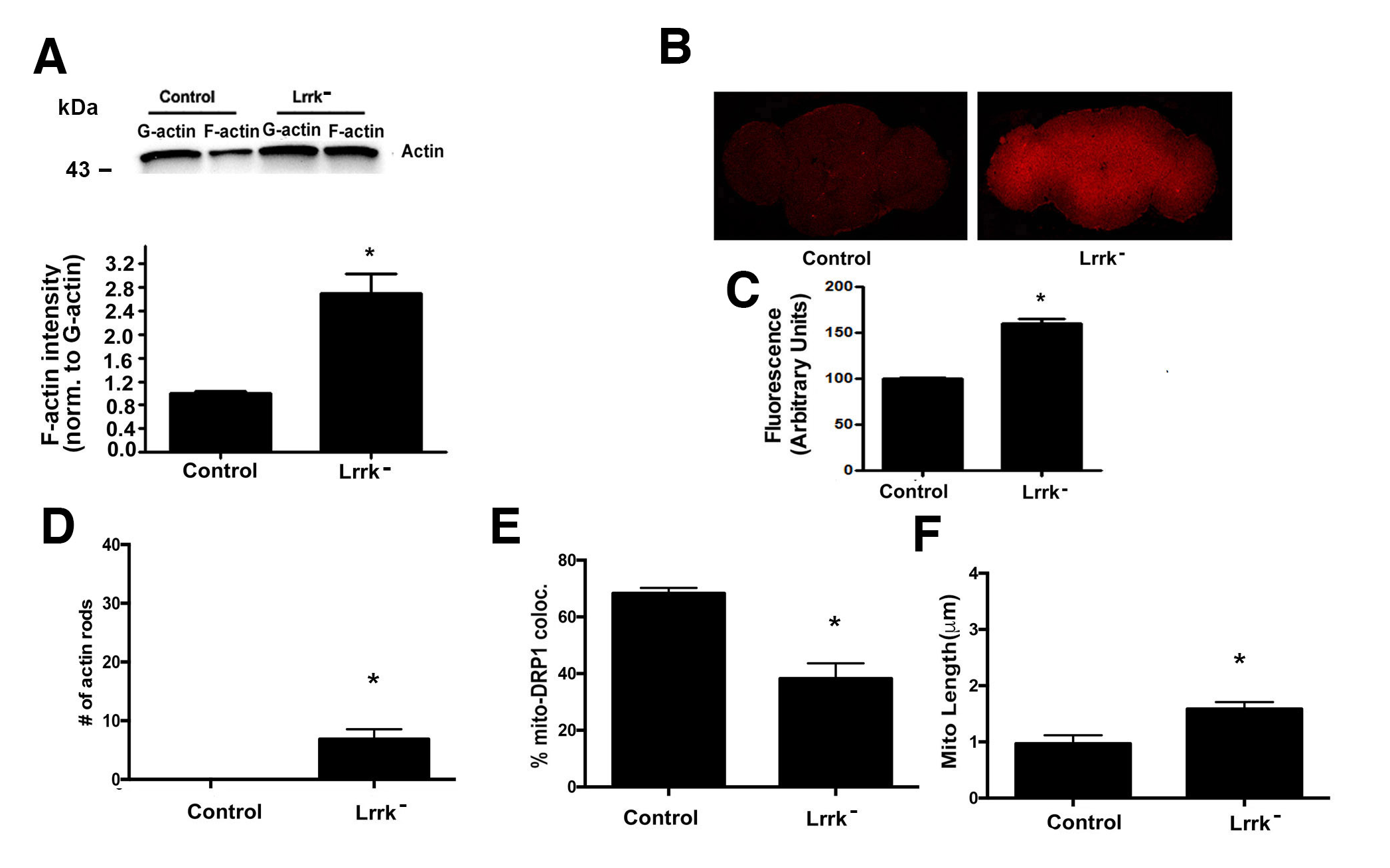

Supplement: S7 Fig — (A) Phalloidin precipitation of F-actin showing increased actin stabilization in flies with loss of Lrrk (Lrrke03680) at 20 days of posteclosion age. Quantification from three separate blots is shown in the lower panel. (B) Representative images of freshly dissected Drosophila brains stained with fluorescent phalloidin. (C) Quantification of the fluorescence intensity of the entire fly brain showing enhanced actin stabilization with loss of Lrrk. (D) Quantification of the number of actin rods in the brains of either control or flies with loss of Lrrk. (E) Quantification of the number of mitochondria colocalized with Drp1 shows reduced mitochondrial localization of Drp1 in flies with reduced Lrrk. (F) Quantification of mitochondrial length shows elongated mitochondria in flies with reduced levels of Lrrk. n = 6 per genotype (B-F). *P < 0.05, t-test. Control is elav-GAL4/+ in A, B, C, and D and elav-GAL4/+; UAS-mito-GFP/+; HA-Drp1/+ in E, F. Flies are 20 days old. See S1 Data for individual numerical values underlying the summary data displayed in A, C—F. Drp1, dynamin-1-like protein; F-actin, filamentous actin; HA, hemagglutinin; Lrrk, leucine-rich repeat kinase; mito-GFP, mitochondrially directed GFP. (TIF) [file pbio.2006265.s007.tif]
