# Supplementary material for: Lrrk promotes tau neurotoxicity through dysregulation of actin and mitochondrial dynamics
Source: PLoS Biol. 2018 Dec 20;16(12):e2006265. doi: 10.1371/journal.pbio.2006265 (PMC6319772; doi:10.1371/journal.pbio.2006265)
Supplement: S1 Data — (DOCX) [file pbio.2006265.s008.docx]

**Individual data Values for Graphs:**

Figure 1A:

| control | tau | tau+Lrrk-null | Lrrk-null | tau+Lrrk-RNAi | Lrrk-RNAi | tau+Lrrk-OE |
| --- | --- | --- | --- | --- | --- | --- |
| 0 | 49 | 96 | 1 | 78 | 1 | 86 |
| 2 | 55 | 92 | 0 | 83 | 1 | 82 |
| 3 | 49 | 85 | 0 | 120 | 0 | 83 |
| 0 | 50 | 85 | 3 | 86 | 0 | 81 |
| 2 | 50 | 89 | 2 | 101 | 1 | 75 |
| 0 | 60 | 78 | 1 | 86 | 0 | 76 |

| Lrrk-OE | tau+Y1383C | Y1383C | tau+ I1915T | I1915T | tau+G1914S | G1914S |
| --- | --- | --- | --- | --- | --- | --- |
| 0 | 118 | 1 | 125 | 0 | 145 | 3 |
| 0 | 125 | 0 | 121 | 0 | 140 | 2 |
| 1 | 106 | 0 | 121 | 0 | 129 | 4 |
| 1 | 120 | 1 | 109 | 2 | 135 | 1 |
| 2 | 106 | 0 | 112 | 1 | 144 | 5 |
| 1 | 96 | 0 | 115 | 0 | 132 | 3 |

Figure 1C

| control | tau | tau+Lrrk- | tau+Lrrk-RNAi | tau+Lrrk-OE |
| --- | --- | --- | --- | --- |
| 0 | 1 | 1.08 | 1.07 | 0.98 |
| 0 | 1 | 0.99 | 0.99 | 1.1 |
| 0 | 1 | 1.02 | 1.03 | 0.97 |

| tau+Y1383C | tau+I1915T | tau+G1914S |
| --- | --- | --- |
| 0.99 | 1.04 | 0.98 |
| 1.01 | 1 | 0.99 |
| 1.02 | 1.01 | 1 |

Figure 1D

| control | tau | tau+Lrrk - | Lrrk - | tau+Lrrk-RNAi | Lrrk-RNAi | tau+Lrrk-OE |
| --- | --- | --- | --- | --- | --- | --- |
| 0 | 45 | 83 | 0 | 79 | 1 | 85 |
| 1 | 42 | 81 | 0 | 75 | 1 | 78 |
| 0 | 51 | 79 | 0 | 74 | 1 | 90 |
| 1 | 41 | 76 | 0 | 79 | 0 | 76 |
| 0 | 35 | 80 | 1 | 79 | 0 | 74 |
| 1 | 43 | 81 | 0 | 80 | 0 | 85 |

| Lrrk-OE | tau+Y1383C | Y1383C | tau+I1915T | I1915T | tau+G1914S | G1914S |
| --- | --- | --- | --- | --- | --- | --- |
| 1 | 96 | 1 | 109 | 1 | 128 | 1 |
| 0 | 116 | 0 | 120 | 1 | 112 | 3 |
| 0 | 105 | 0 | 84 | 0 | 108 | 3 |
| 0 | 92 | 0 | 110 | 0 | 120 | 0 |
| 1 | 98 | 0 | 106 | 0 | 125 | 4 |
| 0 | 96 | 0 | 90 | 0 | 101 | 3 |

Figure 1E:

| control | Lrrk- | Lrrk-RNAi | Lrrk-OE | Y1383C | I1915T | G1914S |
| --- | --- | --- | --- | --- | --- | --- |
| 1 | 0.13 | 0.14 | 2.6 | 2.4 | 2.5 | 2.3 |
| 1 | 0.02 | 0.11 | 1.99 | 1.88 | 1.93 | 1.8 |
| 1 | 0.05 | 0.09 | 1.89 | 1.88 | 1.85 | 1.78 |

Figure 2A

| Ctrl | Tau | Tau+Lrrk-null | Lrrk-null | Tau+Lrrk OE | Lrrk OE | Tau+Lrrk GS | Lrrk GS |
| --- | --- | --- | --- | --- | --- | --- | --- |
| 0.85673 | 5.553591 | 13.01083 | 3.447492 | 15.26236 | 1.69253 | 19.83039 | 1.931794 |
| 1.168531 | 6.507759 | 14.42656 | 2.322266 | 14.41465 | 2.286178 | 18.95669 | 2.177914 |
| 0.867196 | 7.113678 | 12.27102 | 2.092386 | 14.27391 | 3.230964 | 16.62252 | 3.956333 |
| 2.168531 | 3.433779 | 12.6734 | 0.580296 | 14.37423 | 3.556839 | 15.87694 | 3.879105 |
| 1.581739 | 7.526886 | 8.679177 | 1.189462 | 12.90076 | 2.307109 | 15.50956 | 3.245399 |
| 4.784194 | 5.996752 | 11.8856 | 1.455792 | 13.4096 | 3.505233 | 16.69217 | 2.727174 |

Figure 2B

| control | tau | Tau+Lrrk- | Lrrk- | tau+Lrrk-OE | Lrrk-OE | tau+Lrrk-GS | Lrrk-GS |
| --- | --- | --- | --- | --- | --- | --- | --- |
| 1 | 1.01 | 1.15 | 1.21 | 1.1 | 0.99 | 1.1 | 0.98 |
| 1 | 1.112 | 0.98 | 1 | 1.01 | 0.95 | 1 | 0.99 |
| 1 | 1.001 | 0.95 | 0.94 | 0.95 | 1.3 | 0.9 | 1 |

Figure 2D

| Ctrl | Tau | Tau+Lrrk-null | Lrrk-null | Tau+Lrrk-wt | Lrrk-wt | Tau+Lrrk-GS | Lrrk-GS |
| --- | --- | --- | --- | --- | --- | --- | --- |
| 1 | 41 | 79 | 0 | 60 | 3 | 98 | 0 |
| 0 | 42 | 73 | 0 | 75 | 0 | 100 | 0 |
| 0 | 41 | 75 | 1 | 78 | 1 | 97 | 1 |
| 1 | 42 | 70 | 0 | 73 | 0 | 103 | 1 |
| 1 | 45 | 76 | 1 | 78 | 0 | 100 | 2 |
| 0 | 48 | 75 | 0 | 89 | 0 | 104 | 0 |

Figure 2E

| Control | tau | tau,Lrrk-,Lrrk-OE | Lrrk- | Lrrk-OE |
| --- | --- | --- | --- | --- |
| 1 | 1.05 | 1.09 | 0.01 | 1.98 |
| 1 | 1.04 | 0.97 | 0.012 | 2.1 |
| 1 | 0.98 | 0.99 | 0.1 | 2.2 |

Figure 2F

| Control | Tau | Tau+Lrrk-OE+Lrrk-null |
| --- | --- | --- |
| 0 | 53 | 60 |
| 2 | 55 | 58 |
| 3 | 52 | 54 |
| 0 | 50 | 67 |
| 2 | 50 | 45 |
| 0 | 60 | 51 |

Figure 2G

| Control | Tau | Tau+Lrrk-OE+Lrrk-null |
| --- | --- | --- |
| 0 | 40 | 38 |
| 1 | 48 | 50 |
| 0 | 51 | 53 |
| 1 | 41 | 52 |
| 0 | 40 | 48 |
| 1 | 43 | 45 |

Figure 2H

| Control | Tau | Tau+Lrrk-OE+Lrrk-null |
| --- | --- | --- |
| 1 | 41 | 42 |
| 0 | 42 | 48 |
| 0 | 41 | 43 |
| 1 | 42 | 49 |
| 1 | 45 | 50 |
| 0 | 48 | 55 |

Figure 3B

| Control | Tau | Tau+Lrrk-null | Lrrk-null | Tau+Lrrk-OE | Lrrk-OE | Tau+Lrrk-GS | Lrrk-GS |
| --- | --- | --- | --- | --- | --- | --- | --- |
| 1.1 | 1.91 | 2.5 | 0.856122 | 2.8 | 1.104558 | 3.94 | 0.983914 |
| 1.0036 | 1.75 | 2.3 | 1.23291 | 2.29 | 1.042481 | 3.9 | 1.208496 |
| 1.06 | 1.9 | 2.69 | 1.013825 | 2.9 | 0.932719 | 2.98 | 0.968664 |
| 0.86 | 1.8 | 2.99 | 0.890279 | 3.1 | 0.928778 | 3.14 | 1.207892 |
| 0.67 | 2.02 | 2.9 | 1.085433 | 2.8 | 1.108981 | 2.99 | 1.222344 |
| 1.004 | 2.22 | 2.8 | 0.98 | 2.91 | 1.12 | 3.15 | 1.23 |

Figure 3C

| Control | Tau | Tau+Lrrk- | Lrrk- | Tau+Lrrk-OE | Lrrk-OE | Tau+Lrrk-GS | Lrrk-GS |
| --- | --- | --- | --- | --- | --- | --- | --- |
| 66.66 | 25 | 0 | 66.6 | 8 | 66.6 | 3 | 66.6 |
| 70 | 25 | 0 | 75 | 4 | 66.6 | 0 | 70 |
| 66.66 | 37 | 8 | 70 | 5 | 70 | 8 | 70 |
| 70 | 40 | 5 | 66.6 | 0 | 66.6 | 0 | 66.6 |
| 66.66 | 25 | 7 | 65.3 | 5 | 70 | 0 | 66.6 |
| 66.66 | 25 | 10 | 75 | 8 | 66.6 | 5 | 66.6 |

Figure 3D

| Control | Tau | Tau, Lrrk- | Lrrk- | Tau, Lrrk-OE | Lrrk-OE | Tau, Lrrk-GS | LRRK-GS |
| --- | --- | --- | --- | --- | --- | --- | --- |
| 0.6175 | 0.505 | 0.285 | 0.63125 | 0.30125 | 0.6025 | 0.34125 | 0.59 |
| 0.58 | 0.3425 | 0.23 | 0.59625 | 0.20125 | 0.59875 | 0.17875 | 0.63 |
| 0.57125 | 0.44625 | 0.18875 | 0.6425 | 0.225 | 0.57 | 0.17125 | 0.59 |
| 0.6475 | 0.4325 | 0.275 | 0.56375 | 0.18 | 0.6225 | 0.18625 | 0.64 |
| 0.68 | 0.46125 | 0.27375 | 0.65125 | 0.2725 | 0.6275 | 0.20125 | 0.6425 |
| 0.644 | 0.478 | 0.297 | 0.637 | 0.203 | 0.602 | 0.19 | 0.628 |

Figure 3E

| Control | Tau | Tau, Lrrk- | Lrrk- | Tau,Lrrk-OE | Lrrk-OE | Tau, Lrrk-GS | Lrrk-GS |
| --- | --- | --- | --- | --- | --- | --- | --- |
| 0.16625 | 0.447125 | 0.845 | 0.1475 | 0.8925 | 0.19125 | 1.158375 | 0.19625 |
| 0.1725 | 0.45925 | 0.86375 | 0.1475 | 0.88125 | 0.17 | 1.135875 | 0.2125 |
| 0.17625 | 0.445875 | 0.8725 | 0.15125 | 0.99125 | 0.18625 | 1.15425 | 0.19625 |
| 0.165556 | 0.432222 | 0.805556 | 0.147778 | 0.911111 | 0.173333 | 1.206333 | 0.194445 |
| 0.167 | 0.4487 | 0.849 | 0.144 | 0.927 | 0.184 | 1.2647 | 0.218 |
| 0.171111 | 0.444111 | 0.848889 | 0.146667 | 0.947778 | 0.172222 | 1.109333 | 0.196667 |

Figure 3F

| Control | Drp1 | tau | tau+Lrrk- | tau+Lrrk-OE | tau+Lrrk-GS |
| --- | --- | --- | --- | --- | --- |
| 0 | 1 | 0.98 | 0.99 | 0.98 | 0.98 |
| 0 | 1 | 0.95 | 1.1 | 1.2 | 0.97 |
| 0 | 1 | 1.2 | 1.2 | 0.97 | 1.2 |

Figure 3H

| Ctrl | Tau | Lrrk-null | Tau+Lrrk-null | Lrrk-OE | Tau+Lrrk-OE | Lrrk-GS | Tau+Lrrk-GS |
| --- | --- | --- | --- | --- | --- | --- | --- |
| 769.6 | 1139 | 836 | 1567 | 876 | 1754 | 886 | 1996 |
| 901 | 1245 | 601 | 1672 | 997 | 1577 | 765 | 2045 |
| 636 | 1329 | 792 | 1771 | 824 | 1864 | 789 | 1877 |

Figure 3 I

| Ctrl | Tau | Lrrk-null | Tau+Lrrk-null | Lrrk-OE | Tau+Lrrk-OE | Lrrk-GS | Tau+Lrrk-GS |
| --- | --- | --- | --- | --- | --- | --- | --- |
| 0.76 | 1.16 | 0.84 | 2.15 | 0.9 | 1.88 | 0.7 | 2.2 |
| 0.56 | 1.45 | 0.66 | 1.95 | 0.52 | 2.38 | 0.62 | 2.21 |
| 0.56 | 1.5 | 0.6 | 2.1 | 0.71 | 1.85 | 0.66 | 2.4 |

Figure 3J

| Ctrl | tau | Lrrk-null | tau+Lrrk-null | Lrrk-OE | tau+Lrrk-OE | Lrrk-GS | tau+Lrrk-GS |
| --- | --- | --- | --- | --- | --- | --- | --- |
| 100 | 147 | 88 | 255 | 105 | 199 | 95 | 240 |
| 100 | 141 | 120 | 195 | 88 | 243 | 115 | 219 |
| 100 | 155 | 91 | 180 | 98 | 185 | 106 | 192 |
| 100 | 137 | 89 | 175 | 97 | 198 | 99 | 188 |
| 100 | 143 | 75 | 240 | 102 | 198 | 110 | 287 |
| 100 | 135 | 118 | 189 | 89 | 178 | 98 | 245 |

Figure 4A

| Ctrl-Untreated | Ctrl-LatA-5 | Ctrl-LatA-10 | tau-Untreated | tau-LatA-5 | tau-LatA-10 |
| --- | --- | --- | --- | --- | --- |
| 0 | 2 | 4 | 70 | 62 | 14 |
| 0 | 1 | 6 | 67 | 55 | 22 |
| 1 | 0 | 2 | 82 | 54 | 15 |
| 0 | 0 | 2 | 75 | 45 | 18 |
| 0 | 1 | 0 | 62 | 50 | 22 |
| 1 | 0 | 1 | 76 | 52 | 13 |

Figure 4B

| Ctrl-Untreated | Ctrl-LatA-5 | Ctrl-LatA-10 | Untreated | LatA-5 | LatA-10 |
| --- | --- | --- | --- | --- | --- |
| 0 | 0 | 0 | 44 | 29 | 5 |
| 0 | 0 | 0 | 35 | 25 | 3 |
| 1 | 0 | 1 | 43 | 24 | 4 |
| 0 | 1 | 1 | 41 | 28 | 8 |
| 1 | 1 | 0 | 46 | 24 | 6 |
| 0 | 0 | 0 | 47 | 26 | 5 |

Figure 4C

| Ctrl-Untreated | Ctrl-CytoB-5 | Ctrl-CytoB-10 | Untreated | CytoB-5 | CytoB-10 |
| --- | --- | --- | --- | --- | --- |
| 0 | 0 | 0 | 70 | 44 | 8 |
| 0 | 1 | 1 | 67 | 48 | 9 |
| 1 | 1 | 1 | 65 | 55 | 25 |
| 0 | 0 | 3 | 68 | 58 | 35 |
| 0 | 0 | 2 | 59 | 56 | 16 |
| 1 | 1 | 5 | 65 | 53 | 25 |

Figure 4D

| Ctrl-Untreated | Ctrl-5 | Ctrl-10 | Tau-Untr | Tau-5 | Tau-10 |
| --- | --- | --- | --- | --- | --- |
| 0 | 0 | 2 | 44 | 20 | 4 |
| 0 | 0 | 1 | 35 | 15 | 10 |
| 0 | 1 | 1 | 43 | 13 | 2 |
| 1 | 1 | 3 | 41 | 22 | 10 |
| 1 | 3 | 0 | 46 | 25 | 13 |
| 0 | 1 | 0 | 45 | 28 | 5 |

Figure 4E

| Ctrl-Untreated | Ctrl-CytoD-5 | Ctrl-CytoD-10 | Untreated | CytoD-5 | CytoD-10 |
| --- | --- | --- | --- | --- | --- |
| 0 | 0 | 0 | 70 | 50 | 38 |
| 0 | 2 | 3 | 67 | 55 | 12 |
| 1 | 3 | 2 | 65 | 58 | 35 |
| 0 | 0 | 5 | 68 | 62 | 15 |
| 0 | 0 | 0 | 59 | 61 | 40 |
| 1 | 1 | 2 | 65 | 60 | 43 |

Figure 4F

| Ctrl-Untreated | Ctrl-5 | Ctrl-10 | Tau-Untr | Tau-5 | Tau-10 |
| --- | --- | --- | --- | --- | --- |
| 0 | 0 | 2 | 44 | 35 | 16 |
| 0 | 0 | 1 | 35 | 38 | 15 |
| 0 | 1 | 1 | 43 | 29 | 12 |
| 1 | 1 | 3 | 41 | 33 | 10 |
| 1 | 3 | 0 | 46 | 32 | 15 |
| 0 | 1 | 0 | 45 | 35 | 18 |

Figure 4H

| Ctl-Untreated | Ctl-LatA-10 | Ctl-CytoB-10 | Ctl-CytoD-10 | tau-Untreated | tau-LatA-10 | tau-CytoB-10 | tau-CytoD-10 |
| --- | --- | --- | --- | --- | --- | --- | --- |
| 1.26 | 1.38 | 1.06 | 1.5 | 2.9 | 1.2 | 1.2 | 2 |
| 0.91 | 0.93 | 1.98 | 1.23 | 3.4 | 0.45 | 0.7 | 1.4 |
| 0.828 | 1.5 | 1.24 | 1.56 | 4.1 | 2.3 | 2.3 | 2.2 |

Figure 4J

| Ctrl-Untr | Ctrl-LatA | Ctrl-CytoB | Ctrl-CytoD | Tau-Untr | Tau-LatA | Tau-CytoB | TauCytoD |
| --- | --- | --- | --- | --- | --- | --- | --- |
| 1808 | 1869 | 1545 | 1752 | 3653 | 1080 | 2158 | 2258 |
| 1379 | 578 | 1442 | 1645 | 3549 | 1957 | 1825 | 1825 |
| 1520 | 1245 | 1220 | 1772 | 3021 | 943 | 754 | 1725 |

Figure 5C

| control | LRRK2 | tau | tau,LRRK2 |
| --- | --- | --- | --- |
| 445 | 424 | 176 | 173 |
| 441 | 464 | 129 | 129 |
| 402 | 409 | 329 | 107 |
| 424 | 423 | 163 | 150 |
| 389 | 422 | 378 | 137 |
| 452 | 430 | 322 | 148 |
| 441 | 493 | 404 | 310 |
| 461 | 460 | 404 | 388 |
| 463 | 445 | 385 |  |
| 441 | 449 | 301 |  |

Figure 5D

| Control | LRRK2 | Tau | Tau+LRRK2 |
| --- | --- | --- | --- |
| 0 | 0 | 38 | 101 |
| 0 | 0 | 50 | 85 |
| 0 | 0 | 58 | 113 |
| 0 | 0 | 70 | 98 |
| 0 | 0 | 52 | 104 |

Figure 5G

| control | LRRK2 | tau | Tau,LRRK2 |
| --- | --- | --- | --- |
| 0.59 | 0.7 | 0.36 | 0.22 |
| 0.58 | 0.68 | 0.35 | 0.25 |
| 0.62 | 0.67 | 0.33 | 0.23 |
| 0.59 | 0.58 | 0.31 | 0.14 |
| 0.67 | 0.61 | 0.51 | 0.23 |
| 0.58 | 0.7 | 0.45 | 0.21 |
| 0.65 | 0.69 | 0.33 | 0.15 |
| 0.6 | 0.68 | 0.25 | 0.18 |
| 0.59 | 0.64 | 0.37 | 0.21 |
| 0.58 | 0.63 | 0.32 | 0.23 |
| 0.61 | 0.65 | 0.37 | 0.23 |
| 0.68 | 0.63 | 0.5 | 0.14 |
| 0.67 | 0.59 | 0.34 | 0.16 |
| 0.48 | 0.62 | 0.37 | 0.21 |
| 0.59 | 0.61 | 0.36 | 0.22 |
| 0.58 | 0.68 | 0.35 | 0.25 |
| 0.62 | 0.68 | 0.33 | 0.23 |
| 0.59 | 0.69 | 0.31 | 0.14 |
| 0.67 | 0.61 | 0.51 | 0.23 |
| 0.58 | 0.62 | 0.45 | 0.21 |
| 0.65 | 0.57 | 0.33 | 0.15 |
| 0.6 | 0.58 | 0.25 | 0.18 |
| 0.59 | 0.61 | 0.37 | 0.21 |
| 0.58 | 0.57 | 0.32 | 0.23 |
| 0.61 | 0.58 | 0.37 | 0.23 |
| 0.68 | 0.61 | 0.5 | 0.14 |
| 0.67 | 0.6 | 0.34 | 0.16 |
| 0.48 | 0.69 | 0.37 | 0.21 |
| 0.59 | 0.62 | 0.36 | 0.22 |
| 0.58 | 0.58 | 0.35 | 0.25 |
| 0.62 | 0.61 | 0.33 | 0.23 |
| 0.59 | 0.61 | 0.31 | 0.14 |
| 0.67 | 0.59 | 0.51 | 0.23 |
| 0.58 | 0.61 | 0.45 | 0.21 |
| 0.65 | 0.58 | 0.33 | 0.15 |
| 0.6 | 0.59 | 0.25 | 0.18 |
| 0.59 | 0.68 | 0.37 | 0.21 |
| 0.58 | 0.65 | 0.32 | 0.23 |
| 0.61 | 0.63 | 0.37 | 0.23 |
| 0.68 | 0.65 | 0.5 | 0.14 |
| 0.67 | 0.61 | 0.34 | 0.16 |
| 0.48 | 0.59 | 0.37 | 0.21 |

Figure 5H

| control | LRRK2 | tau | tau, LRRK2 |
| --- | --- | --- | --- |
| 1 | 1.03 | 1.54 | 1.93 |
| 1 | 1.05 | 1.59 | 2.02 |
| 1 | 1.1 | 1.65 | 2.23 |
| 1 | 0.99 | 1.68 | 2.06 |
| 1 | 1.1 | 1.44 | 1.92 |

Figure 5I

| control | LRRK2 | tau | tau,LRRK2 |
| --- | --- | --- | --- |
| 0.335 | 0.2975 | 0.405 | 0.9025 |
| 0.2175 | 0.3 | 0.4325 | 0.7825 |
| 0.315 | 0.27 | 0.535 | 0.9025 |
| 0.2875 | 0.31125 | 0.4825 | 0.725 |
| 0.315 | 0.31 | 0.495 | 0.8625 |
| 0.2575 | 0.25625 | 0.4125 | 0.7325 |

Figure 6A

| Time | Actin | | | Actin-LRRK2-WT | | |
| --- | --- | --- | --- | --- | --- | --- |
| 0 | 0 | 0 | 0 | 0 | 0 | 0 |
| 30 | 2.521 | 2.747 | 3.768 | -1.414 | 1.538 | -1.314 |
| 60 | 1.861 | 3.314 | 0.584 | -2.882 | 0.185 | -2.682 |
| 90 | -0.55 | 0.748 | 0.321 | -3.665 | 3.753 | -3.775 |
| 120 | -2.938 | 0.948 | -0.437 | -4.483 | 1.643 | -4.533 |
| 150 | -1.128 | -1.125 | 1.666 | -4.76 | -3.833 | -4.66 |
| 180 | -2.142 | -0.455 | 0.905 | -0.699 | -5.028 | -0.799 |
| 210 | -8.449 | -1.577 | -2.929 | -5.619 | -0.902 | -5.519 |
| 240 | -2.396 | -0.724 | -1.305 | -5.848 | -2.602 | -5.778 |
| 270 | -2.437 | 1.244 | -0.434 | -4.888 | -2.215 | -4.798 |
| 300 | -4 | -1.428 | 2.312 | -7.037 | -2.744 | -6.937 |
| 330 | -6.344 | -2.974 | -1.055 | -9.431 | -5.444 | -8.431 |
| 360 | -2.243 | -3.608 | 0.556 | -8.516 | -5.001 | -9.516 |
| 390 | -4.265 | -2.492 | -0.666 | -9.878 | -2.684 | -9.998 |
| 420 | -5.73 | -1.133 | -3.546 | -10.417 | -5.434 | -8.417 |
| 450 | -5.845 | -3.222 | -1.819 | -14.393 | -7.458 | -13.393 |
| 480 | -2.572 | -4.158 | -4.428 | -11.853 | -6.846 | -11.953 |
| 510 | -4.744 | -3.765 | -2.046 | -10.884 | -4.561 | -11.884 |
| 540 | -8.313 | -4.557 | -1.774 | -11.311 | -4.883 | -12.311 |
| 570 | -7.113 | -2.532 | -1.222 | -14.383 | -6.278 | -13.583 |
| 600 | -6.485 | -1.043 | -2.224 | -15.221 | -6.162 | -15.221 |
| 630 | -4.049 | -3.235 | -3.564 | -13.948 | -9.725 | -13.948 |
| 660 | -8.945 | -3.443 | -3.127 | -13.838 | -9.174 | -12.838 |
| 690 | -7.556 | -3.155 | -2.218 | -13.484 | -11.091 | -14.484 |
| 720 | -9.615 | -4.826 | 0.521 | -14.964 | -11.794 | -12.964 |
| 750 | -6.035 | -3.225 | -1.774 | -16.548 | -13.671 | -15.548 |
| 780 | -6.689 | -0.861 | -1.12 | -13.924 | -10.982 | -11.924 |
| 810 | -9.935 | -3.011 | -4.71 | -20.483 | -12.524 | -16.483 |
| 840 | -7.998 | -2.392 | -0.862 | -16.439 | -14.308 | -16.439 |
| 870 | -8.873 | -3.87 | -3.071 | -18.825 | -13.843 | -17.825 |
| 900 | -11.639 | -3.969 | -4.045 | -15.959 | -14.326 | -14.959 |
| 930 | -4.893 | -3.194 | -6.194 | -18.527 | -13.691 | -18.527 |
| 960 | -9.137 | -4.703 | -3.495 | -17.786 | -15.364 | -16.786 |
| 990 | -11.064 | -0.495 | -3.307 | -19.435 | -11.466 | -15.435 |
| 1020 | -6.22 | -4.538 | -0.969 | -18.594 | -14.392 | -18.594 |
| 1050 | -8.208 | -5.891 | -1.033 | -21.411 | -14.393 | -21.411 |
| 1080 | -10.401 | -5.327 | -1.999 | -18.346 | -15.74 | -18.346 |
| 1110 | -4.926 | -4.867 | -2.222 | -16.051 | -14.628 | -16.051 |
| 1140 | -10.786 | -4.132 | -3.065 | -19.64 | -15.554 | -19.64 |
| 1170 | -7.425 | -2.069 | -2.049 | -19.42 | -18.097 | -19.42 |
| 1200 | -8.302 | -5.499 | -1.171 | -18.428 | -17.404 | -18.428 |
| 1230 | -10.346 | -5.886 | -3.318 | -18.932 | -15.94 | -18.932 |
| 1260 | -8.322 | -6.017 | -4.766 | -20.559 | -21.344 | -20.559 |
| 1290 | -13.023 | -5.992 | -4.437 | -22.409 | -17.821 | -22.409 |
| 1320 | -11.209 | -5.99 | -4.989 | -22.628 | -19.337 | -22.628 |
| 1350 | -9.444 | -4.607 | -1.098 | -19.766 | -21.027 | -19.766 |
| 1380 | -10.986 | -4.344 | -3.784 | -19.907 | -18.917 | -19.907 |
| 1410 | -12.515 | -6.67 | -3.759 | -20.984 | -21.224 | -20.984 |
| 1440 | -15.567 | -5.254 | -4.286 | -23.546 | -20.184 | -23.546 |
| 1470 | -7.723 | -6.136 | -4.03 | -21.685 | -18.997 | -21.685 |
| 1500 | -10.978 | -6.015 | -3.872 | -22.792 | -22.55 | -22.792 |
| 1530 | -13.26 | -6.423 | -3.766 | -22.665 | -23.52 | -22.665 |
| 1560 | -10.069 | -3.891 | -1.789 | -24.887 | -23.764 | -24.887 |
| 1590 | -12.792 | -7.181 | -3.379 | -22.413 | -23.282 | -22.413 |
| 1620 | -9.449 | -3.863 | -2.537 | -23.83 | -20.589 | -23.83 |
| 1650 | -12.929 | -6.139 | -5.159 | -27.077 | -20.68 | -27.077 |
| 1680 | -10.292 | -5.057 | -4.2 | -23.111 | -21.196 | -23.111 |
| 1710 | -11.856 | -7.054 | -4.589 | -24.233 | -23.95 | -24.233 |
| 1740 | -12.096 | -6.902 | -6.168 | -27.515 | -20.146 | -27.515 |
| 1770 | -12.934 | -7.255 | -5.291 | -25.504 | -21.334 | -25.504 |
| 1800 | -11.506 | -5.456 | -4.311 | -27.752 | -28.848 | -27.752 |
| 1830 | -15.398 | -4.39 | -3.149 | -27.434 | -25.334 | -25.434 |
| 1860 | -9.045 | -6.57 | -0.869 | -27.631 | -28.747 | -27.631 |
| 1890 | -10.946 | -8.294 | -3.036 | -25.321 | -29.802 | -25.321 |
| 1920 | -12.275 | -8.067 | -3.204 | -25.115 | -25.989 | -25.115 |
| 1950 | -10.813 | -5.637 | -4.589 | -29.232 | -24.555 | -29.232 |
| 1980 | -13.507 | -4.742 | -4.294 | -26.109 | -25.444 | -26.109 |
| 2010 | -11.498 | -6.701 | -4.774 | -29.378 | -27.91 | -29.378 |
| 2040 | -14.279 | -5.878 | -5.952 | -25.993 | -25.296 | -25.993 |
| 2070 | -12.583 | -5.845 | -3.602 | -29.8 | -26.64 | -29.8 |
| 2100 | -15.07 | -7.3 | -3.479 | -29.657 | -30.503 | -26.657 |
| 2130 | -13.413 | -8.535 | -1.229 | -28.648 | -31.683 | -28.648 |
| 2160 | -12.016 | -7.466 | -4.158 | -27.6 | -30.928 | -27.6 |
| 2190 | -12.794 | -6.096 | -6.23 | -30.86 | -28.112 | -30.86 |
| 2220 | -13.639 | -7.241 | -5.349 | -29.296 | -30.061 | -32.296 |
| 2250 | -18.722 | -7.003 | -2.698 | -35.645 | -35.141 | -33.645 |
| 2280 | -16.869 | -7.729 | -7.441 | -33.019 | -32.424 | -30.019 |
| 2310 | -15.396 | -8.587 | -4.18 | -29.251 | -29.165 | -29.251 |
| 2340 | -11.468 | -8.426 | -5.361 | -31.749 | -29.858 | -31.749 |
| 2370 | -14.578 | -7.392 | -5.23 | -31.497 | -30.644 | -31.497 |
| 2400 | -16.542 | -8.551 | -6.195 | -33.167 | -28.105 | -33.167 |
| 2430 | -14.197 | -7.902 | -6.376 | -33.865 | -31.088 | -27.865 |
| 2460 | -17.55 | -5.917 | -7.253 | -33.403 | -30.839 | -31.403 |
| 2490 | -15.419 | -8.766 | -7.732 | -38.86 | -35.02 | -35.86 |
| 2520 | -14.001 | -8.611 | -2.511 | -33.168 | -31.422 | -34.168 |
| 2550 | -13.481 | -6.897 | -8.92 | -35.669 | -32.779 | -33.669 |
| 2580 | -17.947 | -5.391 | -8.321 | -33.766 | -31.987 | -31.766 |
| 2610 | -14.724 | -7.36 | -6.341 | -36.821 | -35.89 | -34.821 |
| 2640 | -12.725 | -9.492 | -6.256 | -32.723 | -33.102 | -32.723 |
| 2670 | -11.671 | -9.312 | -5.506 | -36.844 | -35.97 | -36.844 |
| 2700 | -13.507 | -8.262 | -7.784 | -32.677 | -35.143 | -32.677 |
| 2730 | -18.396 | -5.888 | -8.149 | -32.493 | -34.434 | -32.493 |
| 2760 | -15.725 | -7.14 | -5.524 | -36.235 | -37.097 | -36.235 |
| 2790 | -15.551 | -6.526 | -6.264 | -32.451 | -35.278 | -32.451 |
| 2820 | -21.511 | -7.706 | -6.239 | -35.718 | -37.248 | -35.718 |
| 2850 | -13.355 | -8.244 | -6.322 | -35.036 | -36.329 | -35.036 |
| 2880 | -17.271 | -8.723 | -5.216 | -34.048 | -35.918 | -34.048 |
| 2910 | -16.349 | -9.44 | -5.792 | -35.272 | -34.985 | -35.272 |
| 2940 | -13.717 | -8.773 | -7.002 | -34.86 | -36.182 | -34.86 |
| 2970 | -17.914 | -8.653 | -6.25 | -34.257 | -35.305 | -36.257 |
| 3000 | -13.088 | -10.042 | -4.232 | -35.787 | -37.461 | -35.787 |
| 3030 | -18.809 | -10.632 | -6.641 | -35.866 | -37.16 | -34.866 |
| 3060 | -14.646 | -10.804 | -7.902 | -36.64 | -36.183 | -36.64 |
| 3090 | -12.725 | -6.34 | -7.784 | -38.456 | -38.886 | -34.043 |
| 3120 | -11.671 | -8.99 | -8.149 | -36 | -38.995 | -35.187 |
| 3150 | -13.507 | -7.55 | -5.524 | -36.761 | -34.245 | -36.096 |
| 3180 | -18.396 | -9.24 | -6.264 | -37.195 | -37.193 | -38.024 |
| 3210 | -19.725 | -9.99 | -6.239 | -37.965 | -36 | -39.548 |
| 3240 | -15.551 | -10.13 | -6.322 | -38.018 | -36.998 | -38.014 |
| 3270 | -21.511 | -12.13 | -5.216 | -38.432 | -37.011 | -41.354 |
| 3300 | -15.355 | -11 | -5.792 | -38.712 | -39.543 | -37.975 |
| 3330 | -17.271 | -12.245 | -7.002 | -38.991 | -38.165 | -40.114 |
| 3360 | -16.349 | -13.22 | -6.25 | -39.176 | -37.243 | -39.012 |
| 3390 | -19.717 | -13.45 | -4.232 | -39.732 | -38.162 | -40.114 |
| 3420 | -17.914 | -16.26 | -6.641 | -41.034 | -39.243 | -41.023 |
| 3450 | -18.088 | -15.87 | -7.902 | -39.721 | -38.974 | -42.114 |
| 3480 | -18.809 | -19.99 | -7.784 | -39.167 | -41.001 | -40.241 |
| 3510 | -19.646 | -18.25 | -8.149 | -40.245 | -39.501 | -39.998 |
| 3540 | -17.035 | -15 | -5.524 | -41.654 | -38.995 | -43.012 |
| 3570 | -18.79 | -18.145 | -6.264 | -41.243 | -39.015 | -39.118 |
| 3600 | -18.79 | -18.145 | -6.264 | -39.214 | -39 | -43.098 |

| Time | Actin-LRRK2-GS | | | Actin-Mix | | |
| --- | --- | --- | --- | --- | --- | --- |
| 0 | 0 | 0 | 0 | 0 | 0 | 0 |
| 30 | 3.699 | 3.261 | -3.083 | -0.865 | 2.499 | 0.841 |
| 60 | 0.783 | 2.007 | -5.297 | -2.994 | -1.456 | -0.424 |
| 90 | -1.054 | 2.164 | -2.134 | -1.293 | -1.125 | -0.576 |
| 120 | 2.412 | 1.297 | -4.173 | -2.096 | -2.73 | -0.262 |
| 150 | -4.686 | -3.47 | -2.513 | -3.471 | -4.024 | -4.07 |
| 180 | -5.004 | -3.164 | -6.83 | -3.299 | -3.189 | -5.084 |
| 210 | -0.264 | -0.782 | -6.227 | -6.844 | -3.853 | -3.474 |
| 240 | -0.995 | -5.069 | -3.899 | -5.017 | -5.531 | -2.828 |
| 270 | -0.52 | -0.437 | -6.305 | -6.773 | -4.412 | -5.096 |
| 300 | -6.406 | -5.575 | -6.428 | -8.708 | -6.215 | -5.59 |
| 330 | -6.642 | -11.896 | -8.932 | -10.176 | -11.737 | -6.861 |
| 360 | -5.976 | -7.092 | -9.121 | -9.088 | -6.161 | -6.077 |
| 390 | -4.437 | -5.881 | -8.14 | -9.435 | -9.203 | -4.731 |
| 420 | -9.368 | -7.593 | -8.756 | -9.714 | -7.164 | -9.022 |
| 450 | -3.547 | -7.227 | -8.174 | -12.589 | -7.29 | -8.974 |
| 480 | -8.199 | -6.727 | -9.237001 | -10.259 | -8.358 | -10.053 |
| 510 | -8.562 | -9.237001 | -7.506 | -11.708 | -8.123 | -9.461 |
| 540 | -10.233 | -10.745 | -8.108 | -11.943 | -4.386 | -8.399 |
| 570 | -10.281 | -10.968 | -9.789 | -8.23 | -7.9 | -9.546 |
| 600 | -13.63 | -13.134 | -12.949 | -12.547 | -8.414 | -12.01 |
| 630 | -16.525 | -11.896 | -11.315 | -13.657 | -8.706 | -14.296 |
| 660 | -14.779 | -12.952 | -15.802 | -10.347 | -6.846 | -10.29 |
| 690 | -15.753 | -17.875 | -12.75 | -15.583 | -11.622 | -13.008 |
| 720 | -14.525 | -15.726 | -14.293 | -12.973 | -10.659 | -14.822 |
| 750 | -15.178 | -15.432 | -12.619 | -13.351 | -7.387 | -11.71 |
| 780 | -16.071 | -14.395 | -14.327 | -13.706 | -10.464 | -13.293 |
| 810 | -18.777 | -16.329 | -14.925 | -14.844 | -10.444 | -13.011 |
| 840 | -14.849 | -13.704 | -15.332 | -17.361 | -9.782 | -12.305 |
| 870 | -17.986 | -17.984 | -15.833 | -18.091 | -12.067 | -16.71 |
| 900 | -17.067 | -15.424 | -17.009 | -17.389 | -15.388 | -18.084 |
| 930 | -19.924 | -19.637 | -16.194 | -17.334 | -14.262 | -15.565 |
| 960 | -16.49 | -18.495 | -14.998 | -18.78 | -15.334 | -17.664 |
| 990 | -17.85 | -18.939 | -17.481 | -17.151 | -15.424 | -14.444 |
| 1020 | -21.194 | -21.46 | -17.613 | -18.373 | -18.045 | -15.407 |
| 1050 | -17.107 | -17.737 | -18.176 | -17.71 | -17.279 | -19.485 |
| 1080 | -23.557 | -19.584 | -20.6 | -16.066 | -17.017 | -20.176 |
| 1110 | -22.908 | -19.8 | -18.965 | -14.578 | -15.974 | -17.944 |
| 1140 | -22.989 | -20.522 | -17.327 | -16.284 | -14.259 | -16.74 |
| 1170 | -24.047 | -19.034 | -18.422 | -14.021 | -15.642 | -20.234 |
| 1200 | -26.999 | -22.528 | -21.318 | -18.33 | -17.607 | -20.31 |
| 1230 | -26.482 | -22.462 | -18.227 | -18.794 | -15.625 | -19.143 |
| 1260 | -25.27 | -21.663 | -20.543 | -16.808 | -18.12 | -20.934 |
| 1290 | -25.902 | -23.161 | -23.002 | -17.289 | -16.741 | -19.316 |
| 1320 | -29.388 | -23.32 | -22.311 | -16.199 | -17.342 | -22.138 |
| 1350 | -28.7 | -23.818 | -22.101 | -17.033 | -18.114 | -21.548 |
| 1380 | -29.139 | -24.14 | -24.045 | -19.7 | -18.5 | -22.211 |
| 1410 | -28.784 | -25.917 | -23.649 | -19.724 | -18.127 | -24.716 |
| 1440 | -24.239 | -26.465 | -22.511 | -20.881 | -19.303 | -21.698 |
| 1470 | -24.983 | -25.45 | -25.265 | -16.144 | -19.282 | -21.544 |
| 1500 | -30.227 | -21.909 | -23.7 | -20.276 | -22.886 | -24.396 |
| 1530 | -27.938 | -30.437 | -25.728 | -17.364 | -22 | -26.799 |
| 1560 | -29.178 | -27.214 | -25.837 | -17.813 | -17.882 | -23.449 |
| 1590 | -29.153 | -30.19 | -26.565 | -20.362 | -22.509 | -23.152 |
| 1620 | -26.475 | -25.759 | -26.357 | -20.742 | -21.856 | -23.518 |
| 1650 | -28.898 | -27.937 | -26.93 | -19.581 | -21.363 | -23.518 |
| 1680 | -30.761 | -24.651 | -28.182 | -17.303 | -21.203 | -23.281 |
| 1710 | -30.669 | -27.345 | -27.763 | -23.609 | -25.211 | -23.602 |
| 1740 | -31.853 | -26.07 | -26.886 | -22.882 | -22.299 | -24.35 |
| 1770 | -32.875 | -26.798 | -28.597 | -23.552 | -23.375 | -25.227 |
| 1800 | -35.942 | -30.074 | -29.862 | -23.152 | -22.72 | -26.384 |
| 1830 | -34.346 | -29.635 | -29.281 | -21.905 | -22.265 | -26.199 |
| 1860 | -33.524 | -27.986 | -28.901 | -21.671 | -22.595 | -25.847 |
| 1890 | -31.508 | -29.309 | -27.65 | -22.615 | -21.131 | -23.121 |
| 1920 | -36.945 | -29.299 | -30.136 | -23.78 | -23.268 | -26.604 |
| 1950 | -31.351 | -28.617 | -28.972 | -23.334 | -23.21 | -23.584 |
| 1980 | -38.328 | -34.37 | -30.789 | -24.652 | -23.081 | -27.907 |
| 2010 | -35.952 | -32.99 | -33.185 | -22.24 | -23.013 | -28.852 |
| 2040 | -35.875 | -29.68 | -31.662 | -22.246 | -24.846 | -26.06 |
| 2070 | -37.021 | -35.16 | -31.426 | -25.311 | -23.09 | -28.663 |
| 2100 | -41.078 | -35.676 | -33.334 | -25.827 | -22.604 | -29.224 |
| 2130 | -39.923 | -34.01 | -34.804 | -24.527 | -27.869 | -29.977 |
| 2160 | -39.774 | -34.122 | -32.987 | -25.187 | -25.821 | -29.882 |
| 2190 | -37.206 | -31.954 | -35.01 | -24.315 | -27.545 | -27.951 |
| 2220 | -39.758 | -40.865 | -36.849 | -26.815 | -31.026 | -30.852 |
| 2250 | -43.824 | -37.562 | -35.93 | -27.32 | -31.636 | -31.848 |
| 2280 | -38.316 | -37.393 | -34.691 | -26.326 | -27.145 | -28.926 |
| 2310 | -37.301 | -39.961 | -36.542 | -27.329 | -26.988 | -31.099 |
| 2340 | -37.206 | -33.356 | -35.701 | -26.231 | -27.909 | -30.819 |
| 2370 | -38.318 | -35.136 | -35.915 | -26.08 | -27.931 | -30.092 |
| 2400 | -39.157 | -37.333 | -38.222 | -23.698 | -31.372 | -29.953 |
| 2430 | -38.191 | -36.264 | -38.748 | -28.531 | -28.205 | -31.92 |
| 2460 | -40.476 | -39.256 | -39.145 | -27.006 | -29.542 | -32.192 |
| 2490 | -41.945 | -37.053 | -37.622 | -26.905 | -27.441 | -33.484 |
| 2520 | -41.423 | -32.687 | -39.018 | -25.738 | -28.772 | -31.555 |
| 2550 | -45.568 | -37.555 | -37.257 | -26.637 | -31.481 | -34.408 |
| 2580 | -43.123 | -36.497 | -38.252 | -26.444 | -29.249 | -31.105 |
| 2610 | -40.034 | -40.908 | -37.849 | -26.749 | -30.248 | -32.851 |
| 2640 | -44.993 | -41.771 | -43.468 | -26.896 | -29.329 | -34.913 |
| 2670 | -42.064 | -39.907 | -39.296 | -26.187 | -32.646 | -32.952 |
| 2700 | -44.058 | -40.391 | -41.434 | -27.573 | -30.49 | -34.734 |
| 2730 | -39.698 | -38.056 | -40.669 | -26.738 | -30.44 | -33.921 |
| 2760 | -42.15 | -40.773 | -42.131 | -28.87 | -32.904 | -30.328 |
| 2790 | -45.593 | -42.202 | -41.532 | -30.466 | -31.477 | -31.215 |
| 2820 | -41.141 | -44.129 | -45.604 | -28.536 | -30.596 | -30.667 |
| 2850 | -42.194 | -39.244 | -41.288 | -28.209 | -31.918 | -30.363 |
| 2880 | -41.792 | -42.948 | -43.137 | -26.861 | -29.642 | -31.645 |
| 2910 | -40.459 | -41.019 | -43.813 | -26.462 | -32.458 | -30.439 |
| 2940 | -42.154 | -42.026 | -44.548 | -29.816 | -32.479 | -35.754 |
| 2970 | -43.136 | -43.77 | -43.917 | -30.131 | -32.897 | -27.086 |
| 3000 | -42.15 | -38.095 | -43.545 | -26.58 | -31.809 | -30.459 |
| 3030 | -45.881 | -42.736 | -44.422 | -31.053 | -29.72 | -29.517 |
| 3060 | -41.22 | -43.021 | -45.306 | -30.015 | -31.372 | -31.242 |
| 3090 | -44.646 | -41.816 | -41.121 | -28.154 | -31.65 | -30.78 |
| 3120 | -43.15 | -40.391 | -43.468 | -23.145 | -27.441 | -30.667 |
| 3150 | -45.881 | -38.056 | -39.296 | -23 | -28.772 | -30.363 |
| 3180 | -44.22 | -40.773 | -41.434 | -30.123 | -31.481 | -31.645 |
| 3210 | -45.646 | -42.202 | -40.669 | -27.573 | -29.249 | -30.439 |
| 3240 | -48.15 | -44.129 | -42.131 | -26.738 | -30.248 | -29.754 |
| 3270 | -47.593 | -39.244 | -41.532 | -28.87 | -29.329 | -27.086 |
| 3300 | -45.141 | -42.948 | -45.604 | -30.466 | -32.646 | -30.459 |
| 3330 | -46.194 | -41.019 | -41.288 | -28.536 | -30.49 | -29.517 |
| 3360 | -48.792 | -42.026 | -43.137 | -28.209 | -30.44 | -31.242 |
| 3390 | -49.459 | -43.77 | -43.813 | -26.861 | -32.904 | -29.78 |
| 3420 | -44.154 | -38.095 | -44.548 | -26.462 | -31.477 | -29.329 |
| 3450 | -49.136 | -42.736 | -43.917 | -29.816 | -30.596 | -32.646 |
| 3480 | -46.15 | -43.021 | -43.545 | -30.131 | -31.918 | -30.49 |
| 3510 | -45.881 | -41.816 | -44.422 | -26.58 | -33.642 | -30.44 |
| 3540 | -45.22 | -43.132 | -45.306 | -31.053 | -32.458 | -32.904 |
| 3570 | -44.132 | -45.231 | -41.121 | -30.015 | -29.237 | -31.765 |
| 3600 | -44.023 | -46.712 | -44.324 | -28.154 | -31.204 | -32.154 |

Figure 6B

| Time | Actin | | | | | Actin-LRRK2-WT | | |
| --- | --- | --- | --- | --- | --- | --- | --- | --- |
| 0 | 0 | | 0 | | 0 | 0 | 0 | 0 |
| 30 | 2.521 | | 2.747 | | 3.768 | -1.414 | 1.538 | -1.314 |
| 60 | 1.861 | | 3.314 | | 0.584 | -2.882 | 0.185 | -2.682 |
| 90 | -0.55 | | 0.748 | | 0.321 | -3.665 | 3.753 | -3.775 |
| 120 | -2.938 | | 0.948 | | -0.437 | -4.483 | 1.643 | -4.533 |
| 150 | -1.128 | | -1.125 | | 1.666 | -4.76 | -3.833 | -4.66 |
| 180 | -2.142 | | -0.455 | | 0.905 | -0.699 | -5.028 | -0.799 |
| 210 | -8.449 | | -1.577 | | -2.929 | -5.619 | -0.902 | -5.519 |
| 240 | -2.396 | | -0.724 | | -1.305 | -5.848 | -2.602 | -5.778 |
| 270 | -2.437 | | 1.244 | | -0.434 | -4.888 | -2.215 | -4.798 |
| 300 | -4 | | -1.428 | | 2.312 | -7.037 | -2.744 | -6.937 |
| 330 | -6.344 | | -2.974 | | -1.055 | -9.431 | -5.444 | -8.431 |
| 360 | -2.243 | | -3.608 | | 0.556 | -8.516 | -5.001 | -9.516 |
| 390 | -4.265 | | -2.492 | | -0.666 | -9.878 | -2.684 | -9.998 |
| 420 | -5.73 | | -1.133 | | -3.546 | -10.417 | -5.434 | -8.417 |
| 450 | -5.845 | | -3.222 | | -1.819 | -14.393 | -7.458 | -13.393 |
| 480 | -2.572 | | -4.158 | | -4.428 | -11.853 | -6.846 | -11.953 |
| 510 | -4.744 | | -3.765 | | -2.046 | -10.884 | -4.561 | -11.884 |
| 540 | -8.313 | | -4.557 | | -1.774 | -11.311 | -4.883 | -12.311 |
| 570 | -7.113 | | -2.532 | | -1.222 | -14.383 | -6.278 | -13.583 |
| 600 | -6.485 | | -1.043 | | -2.224 | -15.221 | -6.162 | -15.221 |
| Time | Actin-LRRK2-GS | | | | | Actin-Mix | | |
| 0 | 0 | 0 | | 0 | | 0 | 0 | 0 |
| 30 | 3.699 | 3.261 | | -3.083 | | -0.865 | 2.499 | 0.841 |
| 60 | 0.783 | 2.007 | | -5.297 | | -2.994 | -1.456 | -0.424 |
| 90 | -1.054 | 2.164 | | -2.134 | | -1.293 | -1.125 | -0.576 |
| 120 | 2.412 | 1.297 | | -4.173 | | -2.096 | -2.73 | -0.262 |
| 150 | -4.686 | -3.47 | | -2.513 | | -3.471 | -4.024 | -4.07 |
| 180 | -5.004 | -3.164 | | -6.83 | | -3.299 | -3.189 | -5.084 |
| 210 | -0.264 | -0.782 | | -6.227 | | -6.844 | -3.853 | -3.474 |
| 240 | -0.995 | -5.069 | | -3.899 | | -5.017 | -5.531 | -2.828 |
| 270 | -0.52 | -0.437 | | -6.305 | | -6.773 | -4.412 | -5.096 |
| 300 | -6.406 | -5.575 | | -6.428 | | -8.708 | -6.215 | -5.59 |
| 330 | -6.642 | -11.896 | | -8.932 | | -10.176 | -11.737 | -6.861 |
| 360 | -5.976 | -7.092 | | -9.121 | | -9.088 | -6.161 | -6.077 |
| 390 | -4.437 | -5.881 | | -8.14 | | -9.435 | -9.203 | -4.731 |
| 420 | -9.368 | -7.593 | | -8.756 | | -9.714 | -7.164 | -9.022 |
| 450 | -3.547 | -7.227 | | -8.174 | | -12.589 | -7.29 | -8.974 |
| 480 | -8.199 | -6.727 | | -9.237001 | | -10.259 | -8.358 | -10.053 |
| 510 | -8.562 | -9.237001 | | -7.506 | | -11.708 | -8.123 | -9.461 |
| 540 | -10.233 | -10.745 | | -8.108 | | -11.943 | -4.386 | -8.399 |
| 570 | -10.281 | -10.968 | | -9.789 | | -8.23 | -7.9 | -9.546 |
| 600 | -13.63 | -13.134 | | -12.949 | | -12.547 | -8.414 | -12.01 |

Figure 6C

| Actin | Actin+LRRK2-wt | Actin+LRRK2-GS | Actin+Mix |
| --- | --- | --- | --- |
| 0.008 | 0.02 | 0.031 | 0.02 |
| 0.009 | 0.021 | 0.024 | 0.022 |
| 0.013 | 0.023 | 0.029 | 0.019 |

Figure 6D

| Actin | Actin+LRRK2-wt | Actin+LRRK2-GS | Actin+Mix |
| --- | --- | --- | --- |
| 1 | 1.770064 | 1.987135 | 1.270832 |
| 1 | 1.758658 | 2.106421 | 1.407107 |
| 1 | 1.919476 | 1.974079 | 1.432058 |

Figure 6F

| LRRK2-GS | LRRK2-WT | Mix |
| --- | --- | --- |
| 3.015 | 4.388 | 7.5 |
| 3.78 | 3.87 | 6.36 |
| 1.175 | 4.535 | 7.099 |

Figure 6H

| Actin | Actin+LRRK2-WT | Actin+LRRK2-GS | Actin+Mix |
| --- | --- | --- | --- |
| 20.15886 | 6.9107 | 3.514667 | 7.1342 |
| 17.15564 | 6.3935 | 4.0166 | 4.710546 |
| 19.1946 | 12.43 | 2.4194 | 7.132 |
| 17.7327 | 7.123 | 3.55 | 3.4577 |
| 18.95 | 10.543 | 2.1345 | 6.13 |

Figure 6J

| Actin | Actin+LRRK2-WT | Actin+LRRK2-GS | Actin+Mix |
| --- | --- | --- | --- |
| 20.15886 | 8.135329 | 5.543 | 18.45321 |
| 22.1564 | 11.09868 | 6.1254 | 16.2319 |
| 23.5419 | 12.43 | 4.119876 | 15.5632 |
| 20.1965 | 10.23416 | 3.67543 | 14.11345 |
| 21.9765 | 10.543 | 5.11987 | 15.11987 |

Figure 6M

| Total Lrrk (%) | Colocalization (%) |
| --- | --- |
| 100 | 43 |
| 100 | 51 |
| 100 | 29 |

S1 Figure A

| control | SCA3 | SCA3, EGFP | SCA3,Lrrk-RNAi | SCA3,Lrrk-OE |
| --- | --- | --- | --- | --- |
| 0.2618026 | 0.1762452 | 0.1326165 | 0.2 | 0.1849057 |
| 0.4365079 | 0.1420765 | 0.118451 | 0.1608187 | 0.1445783 |
| 0.3287037 | 0.1936759 | 0.1660517 | 0.1930836 | 0.1641337 |
| 0.3270677 | 0.1461794 | 0.2083333 | 0.171875 | 0.1981132 |
| 0.259434 | 0.1941392 | 0.1016043 | 0.1895735 | 0.1836735 |
| 0.4671053 | 0.1470588 | 0.1396397 | 0.1923077 | 0.1571429 |

S1 Figure B

| control | tau | tau,Lrrk-OE | 3KD | tau,3KD |
| --- | --- | --- | --- | --- |
| 0 | 49 | 86 | 0 | 78 |
| 2 | 55 | 82 | 2 | 75 |
| 3 | 49 | 83 | 1 | 76 |
| 0 | 50 | 81 | 2 | 76 |
| 2 | 50 | 85 | 0 | 72 |
| 0 | 60 | 86 | 0 | 71 |

S1 Figure C

| control | tau | tau,Lrrk-OE | Lrrk-3KD | tau,Lrrk-3KD |
| --- | --- | --- | --- | --- |
| 0 | 45 | 85 | 0 | 61 |
| 1 | 42 | 78 | 1 | 61 |
| 0 | 51 | 90 | 0 | 61 |
| 1 | 41 | 76 | 1 | 56 |
| 0 | 35 | 74 | 0 | 63 |
| 1 | 43 | 85 | 0 | 72 |

S2 Figure B

| Control | Tau | Tau +Lrrk-null+Lrrk-GS |
| --- | --- | --- |
| 0 | 40 | 47 |
| 1 | 48 | 55 |
| 0 | 51 | 52 |
| 1 | 41 | 48 |
| 0 | 40 | 47 |
| 1 | 43 | 48 |

S2 Figure C

| Control | Tau | Tau +Lrrk-null+Lrrk-GS |
| --- | --- | --- |
| 0 | 53 | 53 |
| 2 | 55 | 56 |
| 3 | 52 | 58 |
| 0 | 50 | 62 |
| 2 | 50 | 65 |
| 0 | 60 | 55 |

S2 Figure D

| Control | Tau | Tau+Lrrk-OE+Lrrk-null |
| --- | --- | --- |
| 1 | 41 | 48 |
| 0 | 42 | 52 |
| 0 | 41 | 46 |
| 1 | 42 | 49 |
| 1 | 45 | 56 |
| 0 | 48 | 55 |

S2 Figure E

| control | tau | LRRK2 | tau, LRRK2 |
| --- | --- | --- | --- |
| 0 | 49 | 0 | 85 |
| 2 | 55 | 2 | 84 |
| 3 | 49 | 3 | 75 |
| 0 | 50 | 1 | 88 |
| 2 | 50 | 2 | 74 |
| 0 | 60 | 0 | 76 |

S2 Figure F

| control | tau | LRRK2 | tau,LRRK2 |
| --- | --- | --- | --- |
| 0 | 45 | 0 | 72 |
| 1 | 42 | 1 | 68 |
| 0 | 51 | 0 | 78 |
| 1 | 41 | 1 | 76 |
| 0 | 35 | 0 | 77 |
| 1 | 43 | 0 | 79 |

S2 Figure G

| control | tau | LRRK2 | tau,LRRK2 |
| --- | --- | --- | --- |
| 0 | 41 | 0 | 79 |
| 1 | 42 | 0 | 75 |
| 0 | 41 | 1 | 70 |
| 1 | 42 | 0 | 66 |
| 0 | 45 | 1 | 76 |
| 1 | 48 | 0 | 72 |

S3 Figure B

| control | tau | Lrrk- | tau+Lrrk- | Lrrk-OE | tau+Lrrk-OE | Lrrk-GS | tau+Lrrk-GS |
| --- | --- | --- | --- | --- | --- | --- | --- |
| 4.808944 | 2.119565 | 5.808944 | 1.318392 | 4.808944 | 1.977587 | 4.408944 | 0.6591958 |
| 5.092298 | 3.351207 | 4.092298 | 0.6476684 | 4.092298 | 1.295337 | 5.092298 | 1.295337 |
| 4.742548 | 2.647254 | 4.742548 | 1.313198 | 4.742548 | 0.6565988 | 5.742548 | 0.6565988 |
| 5.294507 | 2.457002 | 5.294507 | 0.6301197 | 5.294507 | 0.6301197 | 4.39765 | 0.6301197 |
| 5.545287 | 3.270111 | 4.545287 | 1.268231 | 4.545287 | 0.6341154 | 4.545287 | 0.6341154 |
| 5.242464 | 2.812939 | 4.56432 | 1.250782 | 5.642464 | 1.250782 | 5.242464 | 0.6253909 |

S4 Figure A

| Ctrl | Tau | Tau+Lrrk-null | Tau+Lrrk-OE | Tau+Lrrk-GS | Ctrl | Tau | Tau+Lrrk-null | Tau+Lrrk-OE | Tau+Lrrk-GS | Ctrl | Tau | Tau+Lrrk-null | Tau+Lrrk-OE | Tau+Lrrk-GS | Ctrl | Tau | Tau+Lrrk-null | Tau+Lrrk-OE | Tau+Lrrk-GS |
| --- | --- | --- | --- | --- | --- | --- | --- | --- | --- | --- | --- | --- | --- | --- | --- | --- | --- | --- | --- |
| 0 | 59 | 100 | 109 | 143 | 2 | 7 | 25 | 16 | 22 | 2 | 10 | 22 | 23 | 25 | 2 | 15 | 25 | 32 | 42 |
| 0 | 60 | 95 | 98 | 145 | 1 | 8 | 17 | 28 | 21 | 1 | 12 | 23 | 23 | 22 | 2 | 18 | 29 | 35 | 39 |
| 1 | 62 | 99 | 110 | 138 | 1 | 12 | 16 | 15 | 25 | 1 | 15 | 19 | 19 | 25 | 3 | 15 | 30 | 27 | 35 |
| 0 | 58 | 105 | 102 | 133 | 0 | 11 | 15 | 18 | 23 | 0 | 12 | 22 | 20 | 30 | 0 | 14 | 34 | 29 | 43 |
| 0 | 62 | 106 | 98 | 145 | 0 | 8 | 14 | 14 | 25 | 0 | 10 | 23 | 23 | 25 | 2 | 12 | 34 | 35 | 48 |
| 1 | 55 | 101 | 100 | 148 | 1 | 10 | 18 | 15 | 35 | 1 | 11 | 19 | 23 | 28 | 1 | 16 | 38 | 40 | 45 |

S4 Figure B

| Ctrl | Tau | Tau+Lrrk-null | Tau+Lrrk-OE | Tau+Lrrk-GS | Ctrl | Tau | Tau+Lrrk-null | Tau+Lrrk-OE | Tau+Lrrk-GS | Ctrl | Tau | Tau+Lrrk-null | Tau+Lrrk-OE | Tau+Lrrk-GS | Ctrl | Tau | Tau+Lrrk-null | Tau+Lrrk-OE | Tau+Lrrk-GS |
| --- | --- | --- | --- | --- | --- | --- | --- | --- | --- | --- | --- | --- | --- | --- | --- | --- | --- | --- | --- |
| 2 | 45 | 85 | 88 | 125 | 0 | 5 | 16 | 15 | 18 | 3 | 8 | 16 | 17 | 19 | 3 | 19 | 26 | 32 | 20 |
| 2 | 38 | 83 | 79 | 115 | 2 | 6 | 20 | 21 | 19 | 2 | 7 | 20 | 24 | 22 | 2 | 20 | 24 | 21 | 50 |
| 2 | 41 | 82 | 87 | 110 | 1 | 5 | 15 | 12 | 21 | 5 | 7 | 18 | 19 | 25 | 5 | 26 | 20 | 35 | 43 |
| 0 | 41 | 80 | 76 | 118 | 0 | 10 | 10 | 13 | 19 | 0 | 6 | 17 | 10 | 17 | 0 | 15 | 35 | 31 | 55 |
| 4 | 48 | 79 | 72 | 125 | 1 | 6 | 13 | 15 | 21 | 1 | 8 | 15 | 15 | 24 | 1 | 22 | 25 | 33 | 35 |
| 3 | 41 | 83 | 88 | 105 | 0 | 4 | 19 | 25 | 25 | 2 | 7 | 23 | 25 | 32 | 2 | 28 | 44 | 45 | 29 |

S5 Figure C

| control | Lrrk-OE | Lrrk-GS |
| --- | --- | --- |
| 32477.81 | 54283.65 | 92939.88 |
| 22954.78 | 48123.37 | 88765.24 |
| 29456 | 50014.16 | 77216.65 |

S6 Figure B

| Total Mitos (%) | Colocalization (%) |
| --- | --- |
| 100 | 19 |
| 100 | 38 |
| 100 | 32 |

S7 Figure A

| Control | Lrrk- |
| --- | --- |
| \| 0.9 \| \| --- \| | 2.51 |
| \| 1 \|  \| \| --- \| --- \| | \| 3.46 \| \| --- \| |
| \| 1.1 \|  \| \| --- \| --- \| | \| 2.93 \| \| --- \| |
| \| 1 \|  \| \| --- \| --- \| | \| 1.88 \| \| --- \| |

S7 Figure C

| Control | Lrrk- |
| --- | --- |
| 90 | 142.8 |
| 100 | 191 |
| 110 | 286 |

S7 Figure D

| Control | Lrrk- |
| --- | --- |
| 0 | 6 |
| 0 | 5 |
| 0 | 9 |
| 0 | 8 |
| 0 | 5 |
| 0 | 8 |

S7 Figure E

| Control | Lrrk- |
| --- | --- |
| 66.66 | 40 |
| 70 | 45 |
| 66.66 | 33 |
| 70 | 35 |
| 75 | 45 |
| 60 | 35 |

S7 Figure F

| Control | Lrrk- |
| --- | --- |
| 0.8 | 1.6 |
| 1 | 1.7 |
| 1.1 | 1.45 |
| 1.1 | 1.5 |
| 0.9 | 1.4 |
| 1 | 1.45 |
